# Prohibitin plays a critical role in Enterovirus 71 neuropathogenesis

**DOI:** 10.1371/journal.ppat.1006778

**Published:** 2018-01-11

**Authors:** Issac Horng Khit Too, Isabelle Bonne, Eng Lee Tan, Justin Jang Hann Chu, Sylvie Alonso

**Affiliations:** 1 Department of Microbiology and Immunology, Yong Loo Lin School of Medicine, National University of Singapore, Singapore; 2 Immunology Programme, Life Sciences Institute, National University of Singapore, Singapore; 3 Electron Microscopy Laboratory, Life Sciences Institute, National University of Singapore, Singapore; 4 Department of Pediatrics, Yong Loo Lin School of Medicine, National University of Singapore, Singapore; 5 Centre for Biomedical & Life Sciences, Singapore Polytechnic, Singapore; The University of Chicago, UNITED STATES

## Abstract

A close relative of poliovirus, enterovirus 71 (EV71) is regarded as an important neurotropic virus of serious public health concern. EV71 causes Hand, Foot and Mouth Disease and has been associated with neurological complications in young children. Our limited understanding of the mechanisms involved in its neuropathogenesis has hampered the development of effective therapeutic options. Here, using a two-dimensional proteomics approach combined with mass spectrometry, we have identified a unique panel of host proteins that were differentially and dynamically modulated during EV71 infection of motor-neuron NSC-34 cells, which are found at the neuromuscular junctions where EV71 is believed to enter the central nervous system. Meta-analysis with previously published proteomics studies in neuroblastoma or muscle cell lines revealed minimal overlapping which suggests unique host-pathogen interactions in NSC-34 cells. Among the candidate proteins, we focused our attention on prohibitin (PHB), a protein that is involved in multiple cellular functions and the target of anti-cancer drug Rocaglamide (Roc-A). We demonstrated that cell surface-expressed PHB is involved in EV71 entry into neuronal cells specifically, while membrane-bound mitochondrial PHB associates with the virus replication complex and facilitates viral replication. Furthermore, Roc-A treatment of EV71-infected neuronal cells reduced significantly virus yields. However, the inhibitory effect of Roc-A on PHB in NSC-34 cells was not through blocking the CRAF/MEK/ERK pathway as previously reported. Instead, Roc-A treated NSC-34 cells had lower mitochondria-associated PHB and lower ATP levels that correlated with impaired mitochondria integrity. *In vivo*, EV71-infected mice treated with Roc-A survived longer than the vehicle-treated animals and had significantly lower virus loads in their spinal cord and brain, whereas virus titers in their limb muscles were comparable to controls. Together, this study uncovers PHB as the first host factor that is specifically involved in EV71 neuropathogenesis and a potential drug target to limit neurological complications.

## Author summary

A close relative of poliovirus, Enterovirus 71 (EV71) causes Hand, Foot and Mouth Disease and has been associated with neurological complications in young children. The lack of effective therapeutics is largely due to our limited understanding of the mechanisms involved in EV71 neuropathogenesis. Here, using a proteomics approach, we have identified a unique panel of host proteins that were modulated during EV71 infection of motor-neuron cells, which are found at the neuromuscular junctions where EV71 enters the central nervous system. Among the candidate proteins, we focused our attention on prohibitin (PHB), a protein that is involved in multiple cellular pathways and the target of anti-cancer drug Rocaglamide (Roc-A). We demonstrated that cell surface-expressed PHB is involved in EV71 entry into neuronal cells specifically, while mitochondria-associated PHB is required for viral replication. Furthermore, Roc-A treatment of EV71-infected neuronal cells led to significantly lower virus yields. *In vivo*, EV71-infected mice treated with Roc-A survived longer than the vehicle-treated animals and had significantly lower virus titers in their spinal cord and brain. Together, our work uncovers PHB as the first host factor that is specifically involved in EV71 neuropathogenesis and a possible drug target to limit neurological complications.

## Introduction

Enterovirus 71 (EV71) is a non-enveloped, positive-sense, single-stranded RNA virus, and causes hand, foot and mouth disease (HFMD) in humans. Being a close relative of poliovirus, EV71 is deemed as an important neurotropic virus worldwide [1]. Since its first isolation in California in 1969, several major outbreaks have been reported in China, Singapore, Korea, and Japan [2–5]. Although the clinical manifestations are generally mild and self-limiting, including HFMD and herpangina, severe neurological complications have been consistently reported with EV71-associated infections, causing brainstem encephalitis, acute flaccid paralysis, pulmonary edema and cardiopulmonary failure [6,7]. In addition, some patients who have recovered from severe disease have been reported to develop long term neurologic and psychiatric disorders [8]. There are currently no effective prophylactic or therapeutic agents against EV71. Although several vaccines have completed Phase III clinical trials [9], regulatory issues may limit their widespread utilization. In addition, as these vaccine candidates consist of inactivated virus from a single EV71 genotype (C4), cross-protection against other genotypes may be limited [10,11].

The increasing awareness of life-threatening EV71 infections has boosted research in recent years to further understand virus-host interactions and develop effective antiviral strategies [12–17]. However, the neuropathogenesis of EV71 is still poorly understood. Infection occurs when the virus enters the body upon ingestion and/or inhalation. The virus multiplies initially in the alimentary tract mucosa and rapidly reaches the deep cervical and mesenteric lymph nodes via the tonsils and Peyer’s patches [18]. After a short transient systemic dissemination phase, the virus accumulates and actively replicates in muscles where it is believed to infect motor neurons at the neuromuscular junctions. Experimental evidence supports that EV71 migrates to the brainstem via retrograde axonal transport as previously described for its close relative poliovirus [1,2,19–21]. However, the molecular mechanisms involved in EV71 infection of motor neurons to access the central nervous system (CNS) have not been studied. Indeed *in vitro* studies aiming at studying EV71 neurovirulence have employed neuroblastoma cell lines that may not reflect accurately infection in motor neurons. To address this gap, we have recently reported a novel *in vitro* model of EV71 infection in the murine motor neuron cell line NSC-34 [22]. NSC-34 cells originate from the fusion between murine neuroblastoma and spinal cord cells, and possess motor neuron-like properties, such as generation of action potentials and production of acetylcholine [23], therefore making it a relevant model to study the mechanism of EV71 neuropathogenesis. We demonstrated that NSC-34 cells are permissive to EV71 clinical isolates and found that, unlike any other mammalian cell types so far reported, EV71-infected NSC-34 cells do not undergo apoptosis and lysis. Instead we showed that the virus exits the cells via a non-lytic mode, a phenomenon that has also been previously described for poliovirus [21,24,25]. These unique features thus suggested that the infection cycle of EV71 in NSC-34 cells involves host pathways and partners that are likely to be different from those previously identified in other mammalian cell types such as muscle cells and neuroblastoma cells.

In this work, using a proteomics approach coupled with mass spectrometry, we have identified a panel of cellular proteins that were dynamically regulated during EV71 infection of NSC-34 cells. Among the host protein candidates that were up-regulated, we focused our attention on prohibitin (PHB) and characterized its role during EV71 infection in NSC-34 cells. We also demonstrated the importance of PHB during EV71 infection in a symptomatic mouse model of EV71 infection.

## Results

### Dynamic modulation of host proteins during EV71 infection of NSC-34 cells

To identify the host proteins involved in EV71 infection cycle in NSC-34 cells, a 2DE proteomic approach was undertaken. NSC-34 cells were infected with EV71 at M.O.I. 10, and the cell lysates were harvested at 6, 24, 48 and 72 hours for downstream proteomic analysis in which a range of 350–800 spots were resolved.

**Fig 1:**
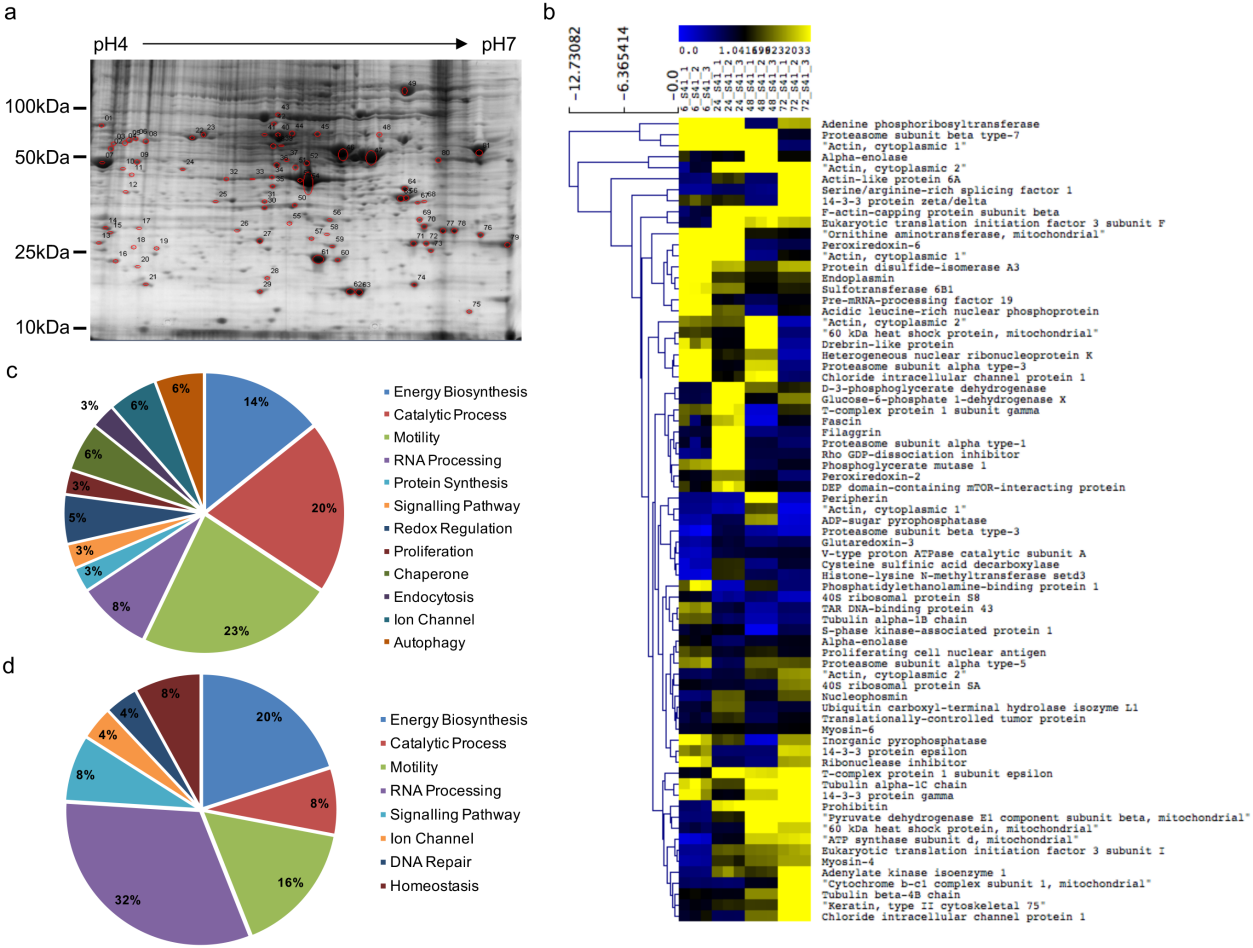
2DE-gel electrophoresis of EV71-infected NSC-34 cells. (a) NSC-34 cells were infected with EV71 at M.O.I. 10 and cell lysate was prepared at 6, 24, 48 and 72 h.p.i. for 2DE gel electrophoresis. Representative 2DE image of infected cell lysate at 48 h.p.i. is shown. Three independent experiments were performed. UI, uninfected. (b) Heat map was generated using MultiExperiment Viewer (MeV). Distance was represented by Euclidean average linkage clustering. The host proteins are further categorized into (c) heavily up-regulated and (d) down-regulated proteins.

By using PDQuest 2-D Analysis Software (BioRad), a total of 81 protein spots (Fig 1a) that displayed at least 0.5-fold differential expression (*p*<0.05, two-tailed Student’s *t*-test) compared to uninfected controls, were excised for in-gel digestion and MALDI-TOF MS analysis. The peptide fingerprints were then searched against NCBInr mouse genome database for protein identification using MASCOT program (http://www.matrixscience.com/). The protein candidates were then categorized based on their primary functional class indicated in UniprotKB database (S1 Table).

To illustrate the dynamic regulation of host proteins during the viral infection, a heat map was generated using MultiExperiment Viewer (MeV), with the distance between proteins represented by Euclidean average linkage clustering (Fig 1b). This clustering analysis revealed that proteins that were up-regulated (Fig 1c) during infection are mainly involved in motility (23%) and catalytic processes (20%), while proteins that participate in RNA processing (32%) and energy biosynthesis (20%) generally displayed a down-regulation trend during the course of infection (Fig 1d).

### *In silico* analysis of the biological function of the host protein candidates

**Fig 2:**
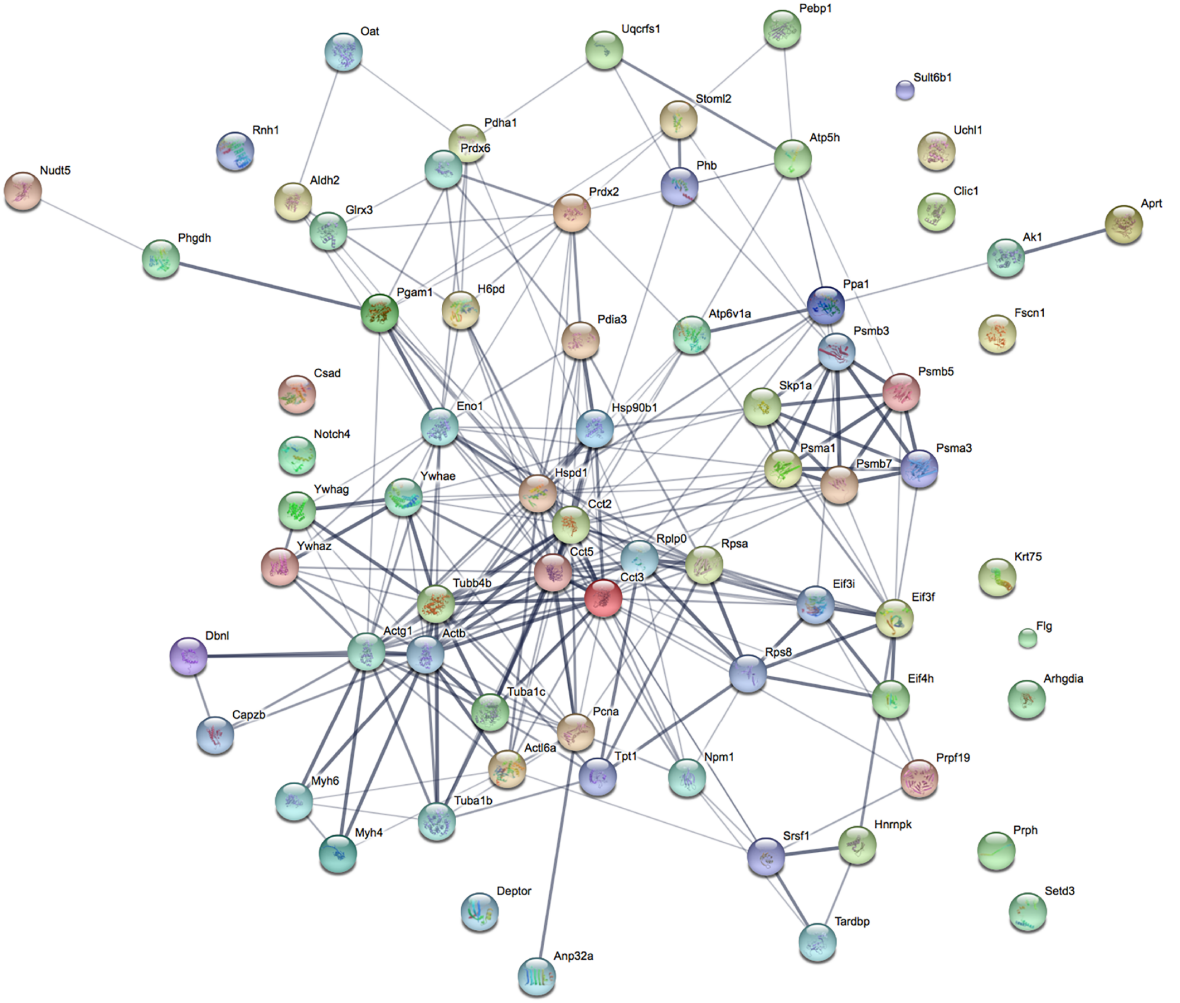
Protein network analysis. The protein network analysis was performed using STRING v10. The confidence level of protein interactions is indicated by the thickness of connecting line. The interactions network is significantly enriched (*p*<0.05). The protein symbols used in this network analysis are listed in S2 Table.

Functional interactions among the selected host proteins were analyzed by STRING (Search Tool for the Retrieval of Interacting Genes/Proteins). This platform allows establish protein-protein interactions based on published literature, online databases, predicted functional associations using genomic information or observations made with other organisms [26]. The protein network obtained was significantly enriched with the *p* value of less than 0.05, suggesting that the interactions are highly associated and unbiased (Fig 2; S2 Table). Furthermore, some of the selected host proteins appear to have strong associations among each other as indicated by the thickness of connecting lines which reflects the confidence level of the interactions [26].

Using GO annotations for biological processes, molecular functions, cellular compartments and protein classes, the protein candidates were localized within the cytoplasm (29.2%), organelles (20.8%) and macromolecular complexes (13.9%) (S1a Fig). In addition, they were found to be involved in various biological processes including mitochondrial biogenesis, proteolytic activity, cytoskeletal machinery and RNA processing (S1a Fig), consistent with the protein clusters observed in the STRING network (Fig 2). Finally, molecular function analysis indicates that majority of these proteins contribute to nucleic acid binding transcription factor activity (34.7%), structural molecule activity (25%) or binding (22.2%) (S1a Fig).

A meta-analysis with other selected proteomic studies of EV71-infected muscle and neuronal cells [13–15,17,27–29] revealed minimal overlap between NSC-34, RD and other neuronal cell types with 4 protein candidates only, namely ACTB, TUBB, PDIA3 and ENO1, suggesting that the host-pathogen interactions in NSC-34 cells are unique (S1b Fig). The limited overlap may also be partly explained by differential proteomics approaches. ACT and TUBB are involved in maintaining cytoskeletal structure, and they are found highly modulated during viral infection to facilitate virus internalization and transportation [30–32]. ENO1 has been shown previously to interact with cytoskeletal proteins in intermediate filaments framework rearrangement [33]. On the other hand, PDI functions in catalyzing reduction and oxidation processes and protein folding [34]. It has also been demonstrated to be involved in humoral immune response [35] or viral replication (DENV) [36] and entry (HIV) [37]. Not surprisingly, greater overlap was seen between motor-neuron NSC-34 and other neuronal cells (26 shared hits) than between NSC-34 and RD cells (5 shared hits). Importantly, neuro-specific proteins such as PRPH and UCHL1 were only identified from profiling studies in NSC-34 and other neuronal cells, thus validating our 2DE proteomic approach.

### Validation of the 2D-proteomics data

Seven protein candidates namely, alpha-enolase (ENO1), DEP domain-containing mTOR-interacting protein (DEPTOR), peripherin (PRPH), phosphatidylethanolamine-binding protein 1 (PEBP1), prohibitin (PHB), stomatin-like protein 2 (STOML2) and protein disulfide-isomerase A3 (PDIA3) were selected for validation of the proteomic findings by gene knockdown. These host proteins have been previously shown to be associated with various steps in the life cycle of viruses, such as entry [37–40] and replication [15,41,42], or to be involved in autophagy [43–45] and axonal transport [46–48]. Silencing of each selected gene target was achieved by reverse transfecting the On-TARGETplus siRNA SMARTpool into NSC-34 cells, prior to viral infection. siRNA SMARTpools consist of four highly potent gene-specific siRNA molecules which have been modified to minimize off-target activity and enhance gene specificity [49].

**Fig 3:**
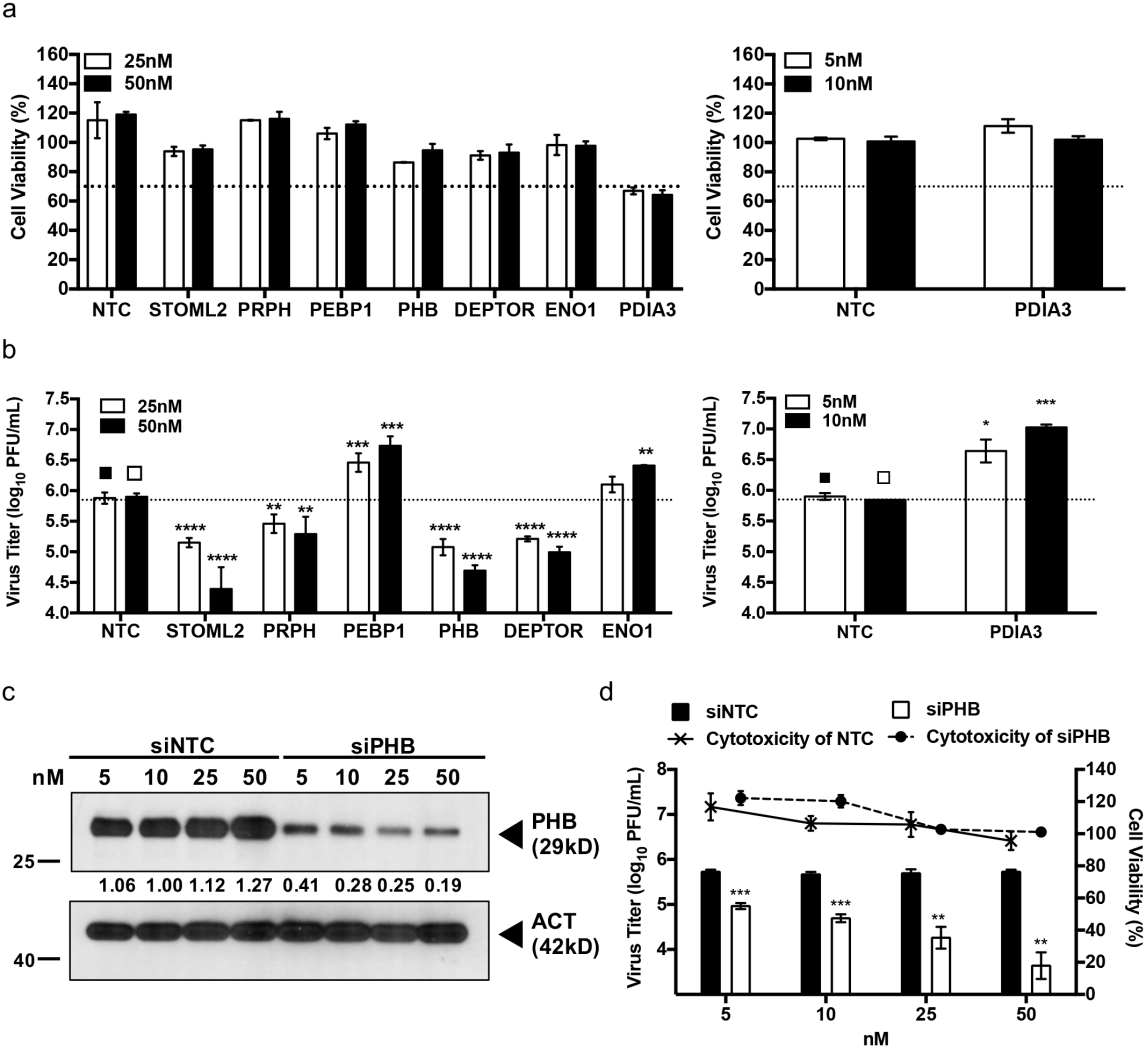
siRNA-mediated gene silencing in NSC-34 cells. NSC-34 cells were transfected with various concentrations of ON-TARGET PLUS siRNA SMARTpools. (a) At 48 h.p.t., the cell viability of transfected cells was assessed using alamarBlue assay. (b) Knocked-down NSC-34 cells were infected with EV71 at M.O.I. 10. The viral titers in the culture supernatant were determined by plaque assay at 48 h.p.i.. Statistical analysis was performed using one-way ANOVA test with Dunnett’s posttest (* *p*<0.1, ** *p*<0.01, *** *p*<0.001, **** *p*<0.0001). (c) NSC-34 cells were transfected with PHB siRNA pool or with non-targeting siRNA (NTC) control at various concentrations. The efficiency of siRNA knockdown was verified at 48 h.p.t. by Western blot. (d) PHB siRNA- or siNTC-transfected cells were infected with EV71 at M.O.I. 10. The viral titers in the culture supernatants were determined by plaque assay at 48 h.p.i.. Cell viability of the transfected cells was assessed using alamarBlue assay. Statistical analysis was performed using two-tailed student’s t-test (** *p*<0.005, *** *p*<0.005). Relative band quantification (below Western blot) was determined by ImageJ, by normalizing to loading control, β-actin. Error bars represent mean ± standard deviation. One representative of two biological repeats is shown.

Cytotoxicity of the siRNAs SMARTpools was first established. Apart from the siRNA pool targeting PDIA3, no significant cytotoxicity was observed with the other siRNA pools at 25 and 50 nM with cell viabilities greater than the 70% viability threshold (Fig 3a). The PDIA3-specific siRNA pool concentrations were lowered to 5 and 10nM to avoid cytotoxicity (Fig 3a). Virus titers in the culture supernatants of siRNA-transfected cells were then determined at 48 h.p.i. Results indicated that silencing of STOML2, PRPH, PHB and DEPTOR led to significantly lower virus titers, whereas PDIA-, PEBP1- and ENO1-knocked down resulted in increased viral titers in the supernatants of EV71-infected NSC-34 cells compared to control (Fig 3b). Importantly, both trends were dose-dependent. Therefore, these results validate the 2D-proteomics approach as a powerful way to identify host proteins that play a role during EV71 infection in NSC-34 cells.

### PHB is involved in virus production

Prohibitins belong to a highly conserved protein family present in unicellular and multicellular eukaryotes [50]. Prohibitin (PHB; BAP-32) and prohibitin 2 (PHB2, REA, BAP-37) are two highly homologous members of this family and are ubiquitously expressed in multiple cellular compartments including the mitochondria, nucleus, and the plasma membrane. Prohibitins have been involved in multiple cellular functions including cell proliferation and maintenance of the functional integrity of the mitochondria [50]. In addition, PHB specifically has been previously reported to be involved in the entry step of alphavirus chikungunya (CHIKV) [40], and flaviviruses Dengue (DENV) [38] and Hepatitis C (HCV) [39], and to interact with envelope proteins from white spot syndrome virus to prevent infection [51]. However, there has been no report so far on the role of PHB during EV71 infection. First, modulation of PHB expression during EV71 infection in NSC-34 cells was confirmed by western blot and showed an overall up-regulation of PHB during the course of infection compared to uninfected control (S2 Fig). Next, the impact of PHB gene silencing on virus production was further analyzed using a wider range of siRNA pool concentrations including 5, 10, 25 and 50 nM. Efficacy of the gene silencing was assessed by Western blot and showed a dose-dependent decrease of PHB expression in NSC-34 cell lysates (Fig 3c). This dose-dependent PHB knockdown correlated well with a dose-dependent reduction in the viral titers measured in the culture supernatant (Fig 3d), therefore supporting the role for PHB in EV71 infection cycle. To address the possibility of false positive or off-target effects of the siRNA pool, PHB gene silencing was performed with the individual siRNAs species from the pool. Western blot showed that each individual siRNA was capable of silencing PHB expression significantly (S3a Fig) which correlated with reduced viral titers measured in the culture supernatants of EV71-infected NSC-34 cells (S3c Fig) with minimal cytotoxicity (S3b Fig). Finally, to further examine the role of PHB during EV71 infection cycle, PHB was over-expressed in NSC-34 cells. Western blot confirmed the upregulation of PHB by 2.6 fold compared to controls (S3d Fig). A significantly higher viral titer was observed in the culture supernatant of PHB over-expressing cells compared to controls (S3e Fig), thus further demonstrating the involvement of PHB in virus production in NSC-34 cells.

### PHB is involved in EV71 entry into NSC-34 cells

**Fig 4:**
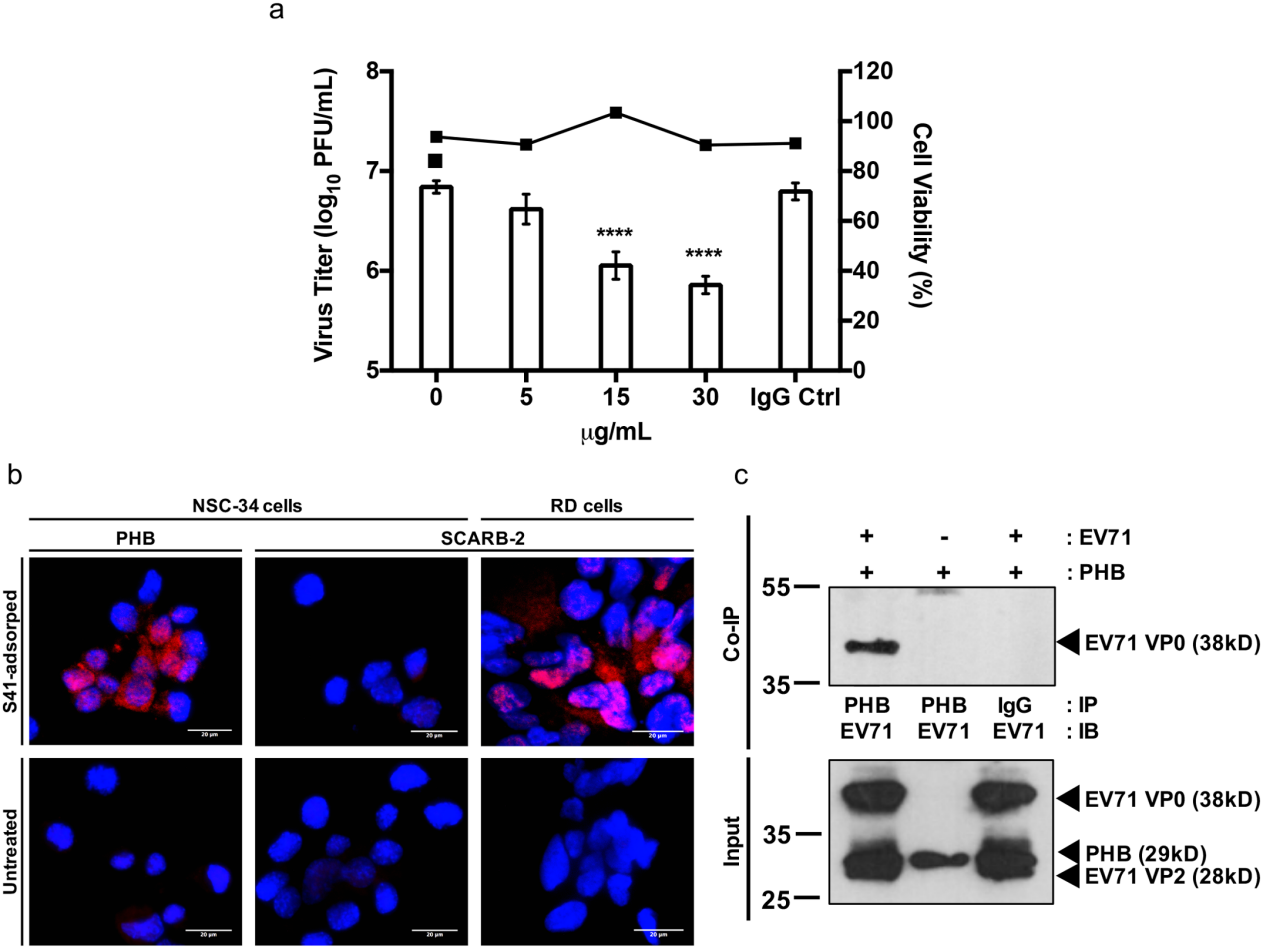
Involvement of PHB in virus entry. (a) Blocking antibody assay. NSC-34 cells were pre-treated with anti-PHB or IgG isotype antibodies, followed by EV71 infection at M.O.I. 10. Culture supernatants were harvested at 48 h.p.i. for viral titer determination by plaque assay. Cell viability was assessed by alamarBlue assay. Statistical analysis was performed using one-way ANOVA with Dunnett’s post-test (***, *p*<0.0005; ****, *p*<0.0001). A representative experiment of two independent repeats is shown. (b) Proximity ligation assay. NSC-34 (M.O.I. 20) and RD cells (M.O.I. 5) were incubated with EV71 at 4°C for 2 hours to allow viral adsorption, prior to PLA staining. SCARB2-stained NSC-34 and RD cells served as negative and positive controls, respectively. Scale bar represents 20μm. A representative experiment of two independent repeats is shown. (c) Co-immunoprecipitation of EV71 and surface-expressed PHB. EV71 and NSC-34 cells were co-incubated for 2 hours at 4°C. The cell lysate was pulled down with anti-PHB or IgG isotype control antibodies prior to immunoblotting using anti-VP1 primary antibody. Mock-infected cells and IgG isotype served as control. A representative experiment of two independent repeats is shown. IP, immunoprecipitation; IB, immunoblot.

To determine if PHB mediates entry of EV71 into NSC-34 cells, a competition assay was performed using commercially available anti-PHB antibody. Incubation of NSC-34 cells with anti-PHB antibody prior to infection led to significant reduction of the viral titer in a dose-dependent manner (Fig 4a). To demonstrate a physical interaction between cell surface-expressed PHB and EV71, a proximity ligation assay (PLA) was performed. In this assay, PHB and EV71 are recognized by specific primary antibodies raised in two different species, which are in turn recognized by species-specific secondary antibodies conjugated to a probe and a target, respectively. Should EV71 and PHB be in close proximity, the probe and the target ligate, amplify and result in emission of a fluorescent signal. SCARB-2 which has been previously demonstrated as the main receptor for EV71 in RD cells [52] was used as positive control and a red fluorescent signal was readily detected in EV71-infected RD cells (Fig 4b). A positive signal was also detected with NSC-34 cells incubated with anti-PHB and anti-EV71 antibodies thus supporting the close proximity between EV71 virus particles and surface-expressed PHB (Fig 4b). In contrast, and expectedly, no signal was detected with mouse SCARB-2 (mSCARB2) and EV71 antibodies (Fig 4b), since we have shown previously that entry of EV71 into NSC-34 cells is not mediated by mSCARB-2 [22].

The physical interaction between EV71 and cell surface-expressed PHB was further assessed by performing a co-immunoprecipitation experiment. NSC-34 cells were incubated with EV71 for 2 hours at 4°C to allow viral adsorption onto the cell surface but no internalization. The total cell lysate was obtained and a pulldown was carried out using antibody specific to PHB or an isotype IgG antibody control. The immunoprecipitates were then analyzed by Western blot using anti-EV71 primary antibody. A discrete band at the expected size was obtained when pulldown was performed with the anti-PHB antibody whereas no band was seen when pulldown was done with the IgG isotype (Fig 4c). These findings thus support that EV71 physically interacts with cell surface-expressed PHB, and suggest that PHB may serve as a receptor for EV71 entry into NSC-34 cells.

### PHB plays a role in the intracellular life cycle of EV71

**Fig 5:**
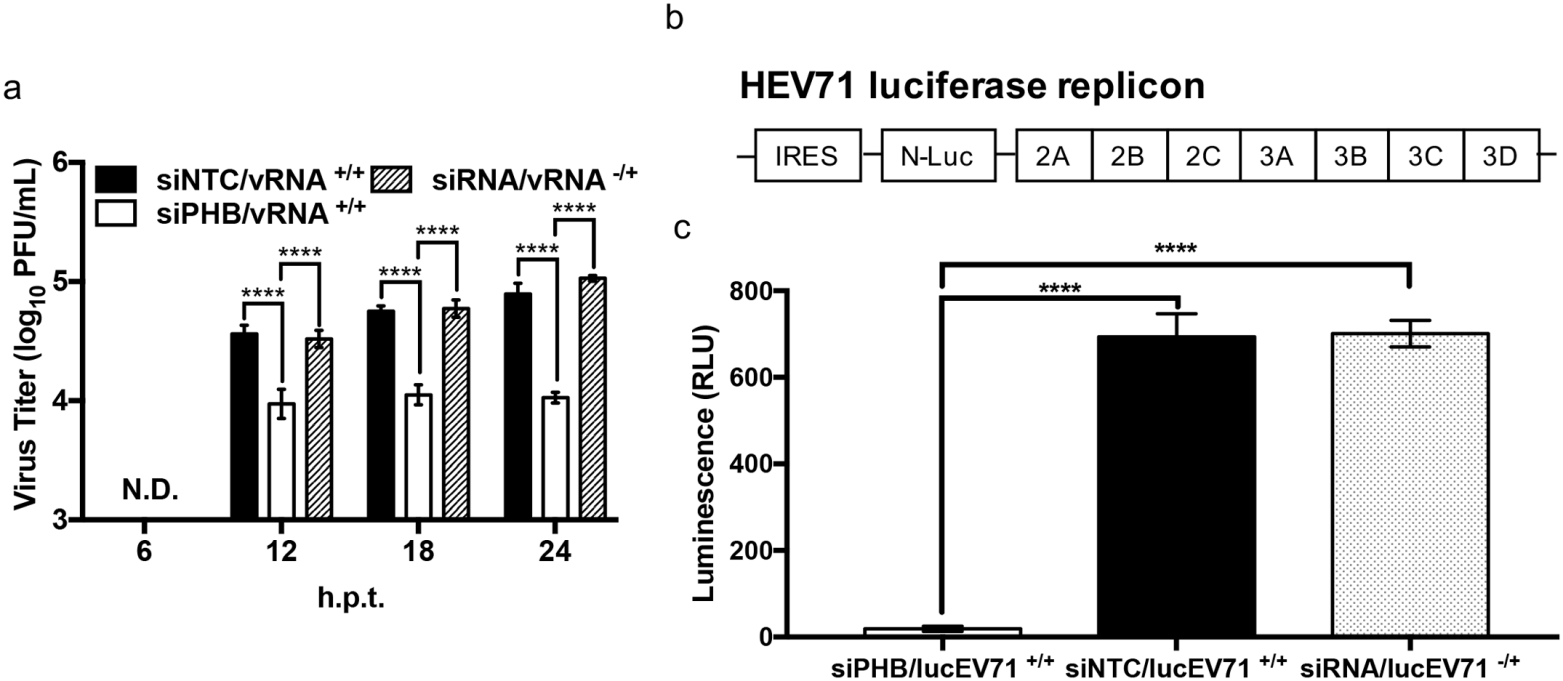
Involvement of intracellular PHB in viral genome replication. (a) NSC-34 cells were reverse-transfected with 50 nM of PHB siRNA, followed by transfection with EV71 purified RNA genome. Culture supernatant was harvested at the indicated time points post-transfection for viral titer determination by plaque assay. Statistical analysis was performed using two-way ANOVA test with Sidak’s multiple comparisons test (****, *p*< 0.0001). Viral RNA only transfection served as control. A representative experiment is shown from two independent repeats. (b) Schematic drawing of lucEV71 replicon. (c) NSC-34 cells were reverse-transfected with 50 nM of PHB siRNA, siNTC or left untreated prior to transfection with 1 μg of lucEV71 RNA. Luminescence signal was read at 48 h.p.t. Statistical analysis was performed using one-way ANOVA with Dunnet’s post-test (****, *p*<0.0001). Error bars represent mean ± standard deviation. One representative of two biological repeats is shown.

In addition to its association to the plasma membrane at the cell surface, PHB is also present intracellularly [50]. To study the role of intracellular PHB in EV71 infection cycle, PHB-knocked down NSC-34 cells were transfected with EV71 RNA genome and the viral titers were determined at 6, 12, 18 and 24 hours post-transfection, in order to assess virus production within a single cell infection cycle. Transfection of viral genome was meant to bypass the virus entry step which we have shown involves cell surface-expressed PHB. No viral titer was obtained at 6 h.p.t. in the PHB-knocked down and control cells (Fig 5a). From 12 h.p.t onwards the viral titers detected in the culture supernatant from PHB-knocked down cells were consistently lower than those measured in siNTC or non-treated cells (Fig 5a). This result thus demonstrates that PHB plays a role in the intracellular virus infection cycle.

To further confirm this hypothesis, a luciferase EV71 (lucEV71) replicon transfection assay was performed. In this replicon, the viral structural genes have been replaced with a luciferase-encoding gene while the other parts of the viral genome are retained (Fig 5b). Upon transfection, the replicon undergoes a single replication cycle with no production of virus progeny since it is deficient in viral structural proteins. The luminescence measured from the cell culture is proportional to the amount of luciferase produced inside the cell thereby reflecting the replication activity of the replicon. Here, a significant reduction in the luminescence signal was observed in PHB-knocked down NSC-34 cells compared to siNTC-treated and non-treated cells (Fig 5c).

Thus, together the data support that intracellular PHB is involved in EV71 viral replication.

### Intracellular PHB interacts with EV71 3D and 3CD non-structural proteins

**Fig 6:**
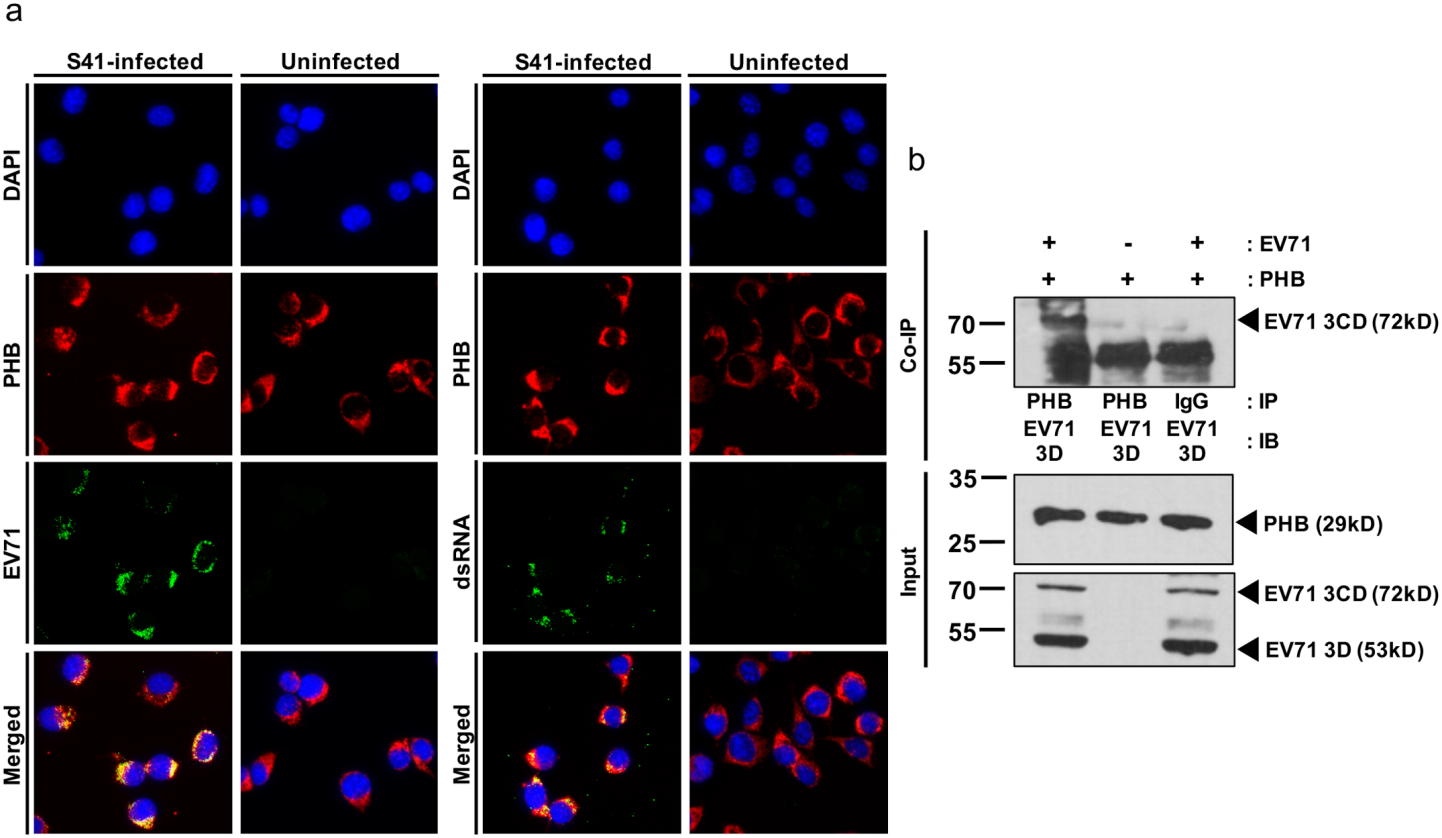
Intracellular PHB co-localizes with EV71 replication complex. (a) NSC-34 cells were infected with EV71 at M.O.I. 10 and fixed with methanol at 48 h.p.i. prior to immunostaining. Uninfected cells served as control. Scale bar represents 20μm. (b) Total cell lysate of infected NSC-34 cells was pulled down with anti-PHB or IgG isotype control antibodies prior to immunoblotting. Mock-infected cells and IgG isotype served as control. IP, immunoprecipitation; IB, immunoblot. One representative of two independent experiments is shown.

To further investigate the role of intracellular PHB during EV71 infection cycle, immunostaining was performed on EV71-infected NSC-34 cells probing for PHB, EV71 capsid proteins VP0/VP1 and the viral replication intermediate dsRNA. Co-localization between PHB and dsRNA, and between PHB and EV71 capsid proteins was readily observed (Fig 6a), supporting that intracellular PHB could be involved in the viral replication and/or assembly processes.

To further study the role of intracellular PHB in viral replication, co-immunoprecipitation was carried out using antibody against PHB. Pull down with anti-PHB antibody followed by Western blot using anti-EV71 3D/3CD antibody led to the detection of a 72kD band that corresponds to the EV71 3CD protein complex and a 53 kDa band (3D polymerase) which co-migrated with IgG heavy chain (Fig 6b). Taken together, these data support a physical interaction between intracellular PHB and EV71 non-structural proteins 3D and 3CD, indicating that intracellular PHB is likely involved in viral replication.

### Mitochondria are exploited as viral replication site in NSC-34 cells

**Fig 7:**
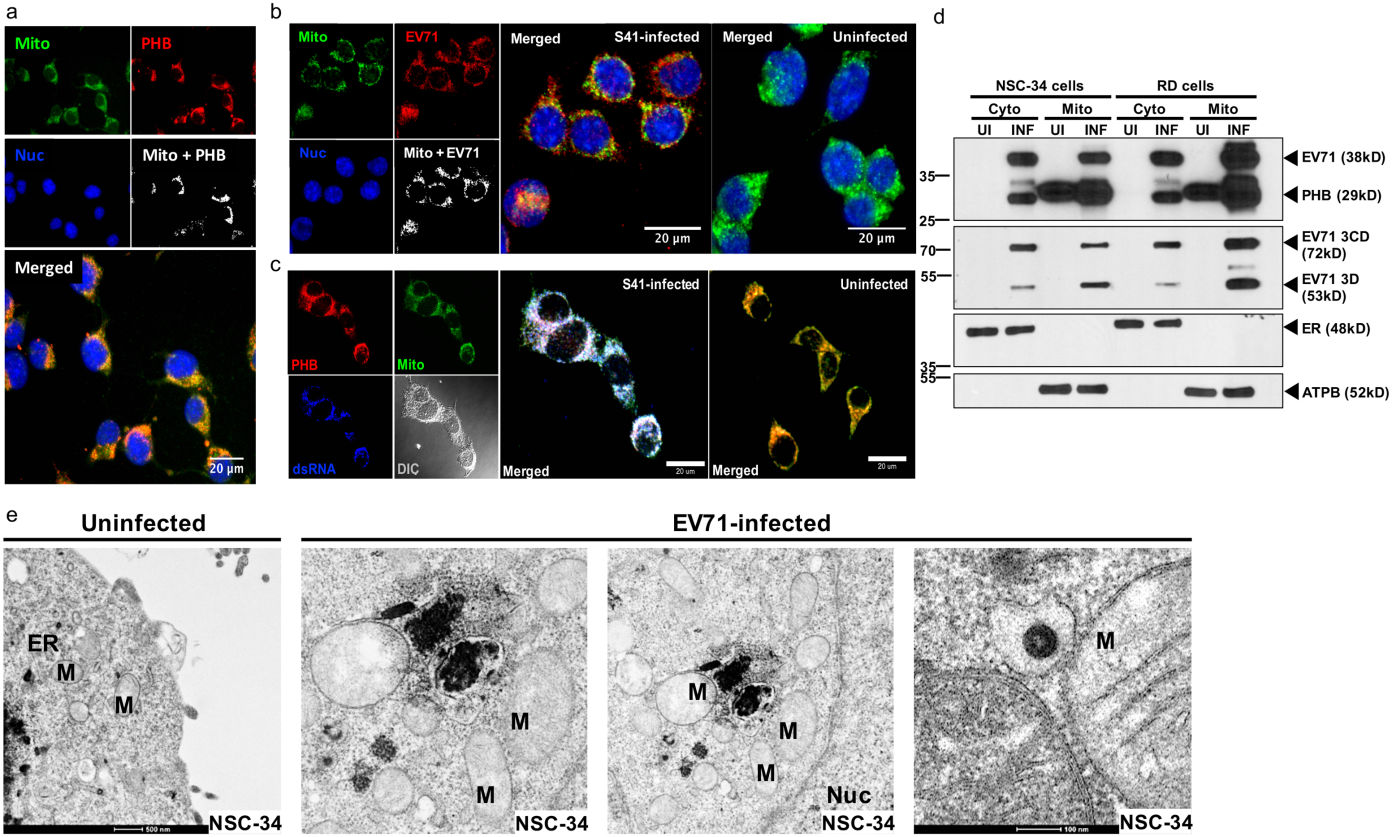
Involvement of mitochondrial PHB in viral replication. (a-c) NSC-34 cells were seeded onto coverslips and infected with EV71 at M.O.I. 10. At 48 h.p.i., the cells were fixed using ice cold methanol and subjected to immunostaining using specific antibodies. Images were post-processed using ImageJ to reveal the co-localization signal. Scale bar represents 20 μm. (d) NSC-34 and RD cells were infected with EV71 at M.O.I. 10 and 1, respectively. At 48 h.p.i. (NSC-34 cells) and 12 h.p.i. (RD cells), the cells were lysed, and the mitochondrial fraction was subjected to Western blot analysis. UI, uninfected; INF, EV71-infected. (e) TEM of EV71-infected NSC-34 cells. NSC-34 cells were infected with EV71 at M.O.I 20 for 48 hours and processed for TEM analysis. M, mitochondria; Nuc, nucleus; V, virus. One representative of two independent experiments is shown.

Previous studies have shown that the main replication sites of picornavirus are located at the Golgi apparatus and endoplasmic reticulum (ER) [53]. We have demonstrated that intracellular PHB co-localizes with dsRNA and is closely associated with the EV71 3D polymerase. Given that intracellular PHB is abundantly and mainly expressed on mitochondria [54], we speculated that in NSC-34 cells mitochondria may be exploited by EV71 as replication site. Consistently, co-localization of PHB and EV71 with mitochondria was observed by IFA (Fig 7a and 7b). Furthermore, co-localization of PHB, dsRNA and mitochondria was also readily detected, thus indicating that mitochondrial PHB is associated with the viral replication complex (Fig 7c). To exclude the possibility that the replication complexes detected were actually associated to the ER, which is in close proximity to mitochondria, the mitochondrial fraction was prepared from EV71-infected NSC-34 cells and Western blot analysis revealed the presence of viral capsid protein VP1 (38 kD), 3D (53 kD) and 3CD (72 kD) proteins (Fig 7d). Furthermore, the mitochondrial fraction was shown to be free of cytoplasmic contamination, as evidenced by the presence of mitochondrial marker (ATPB, 52 kD) and lack of ER marker (Calreticulin, 48 kD). Similar observation was made with the mitochondrial fraction prepared from EV71-infected RD cells (Fig 7d), suggesting that EV71 is able to exploit various cellular organelles as replication scaffolds in various mammalian cell types from different species. This finding is consistent with a previous study where EV71 VP1 was found to be associated with mitochondria in Hela cells [55]. To further support the close proximity and likely interactions between the viral replication complexes and mitochondria, transmission electron microscopy was performed on EV71-infected NSC-34 cells. Clustering of mitochondria surrounding viral replication complexes could be seen (electron-dense like structures) in the infected cells, and examination at a higher magnification indicated a close association between mitochondria membrane and virus complex/virus particle (Fig 7e).

Collectively, the data strongly indicate that mitochondria in NSC-34 cells are exploited by EV71 as a replication scaffold and that mitochondria-associated PHB plays a role in this process.

### Rocaglamide (Roc-A) inhibits EV71 infection of NSC-34 cells

**Fig 8:**
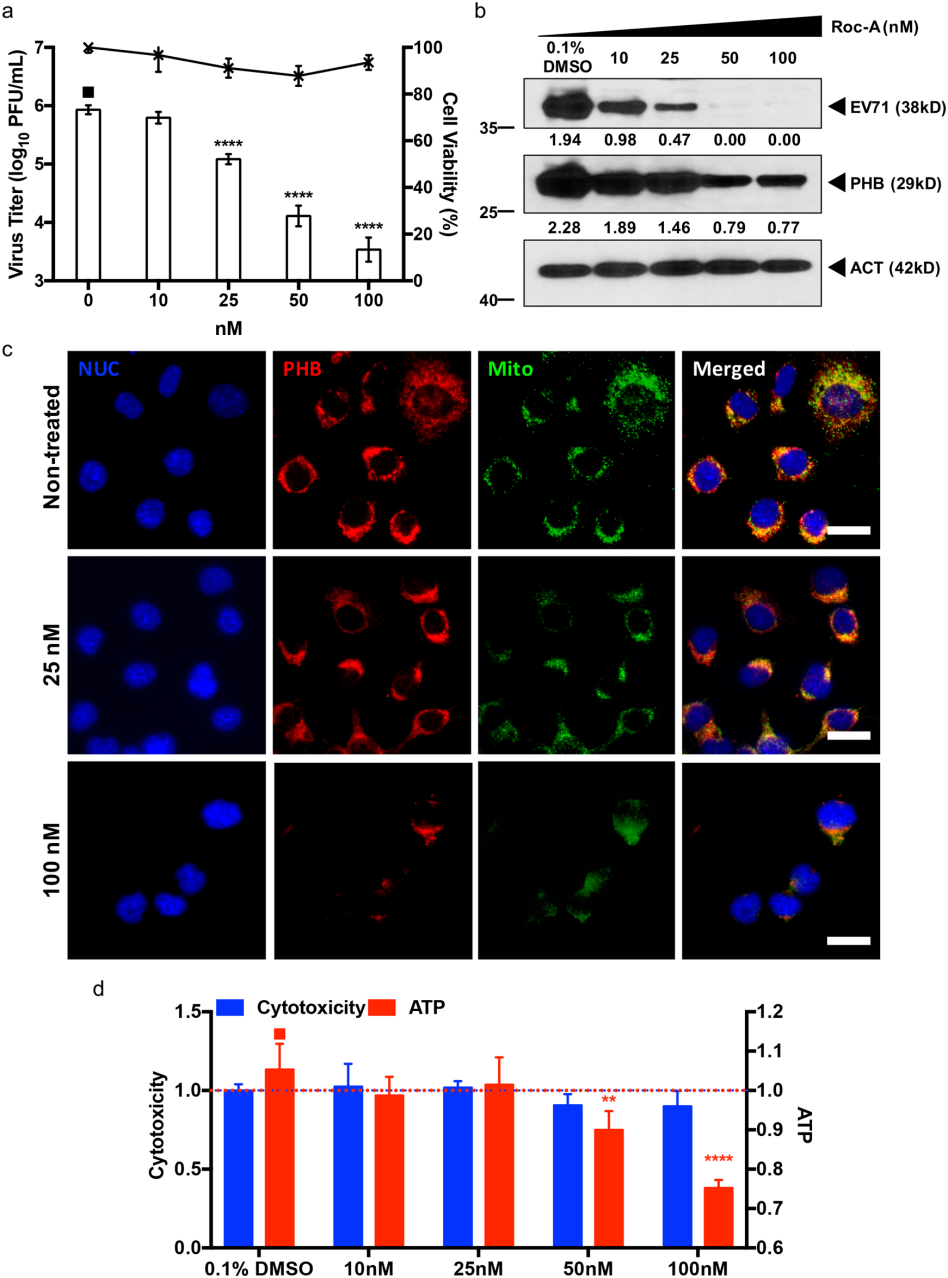
Roc-A treatment of EV71-infected NSC-34 cells. NSC-34 cells were first infected with EV71 at M.O.I. 10, followed by Roc-A treatment for 48 hours. At 48 h.p.i. the culture supernatant was collected for viral titer determination by plaque assay (a). Furthermore, the cell lysate was prepared for Western blot analysis (b). Relative band quantification (below Western blot) was determined by ImageJ, by normalizing to loading control, β-actin. Cell viability was assessed using alamarBlue assay. (c) Immunostaining of Roc-A treated NSC-34 cells. At 48 hours incubation the cells were fixed with ice cold methanol and probed with specific antibodies. Scale bar denotes 20 μm. (d) Mitotoxicity of Roc-A in NSC-34 cells. Cells were incubated with various concentrations of Roc-A for 48 hours before assessment of cytotoxicity (fluorescence) and mitotoxicity (luminescence) using Mitochondrial ToxGlo Assay. Statistical analysis was performed using one-way ANOVA with Dunnett’s post-test (*, *p*<0.05; **, *p*<0.005; ***, *p*<0.001; ****, *p*<0.0001). One representative from two independent experiments is shown.

Recent studies have shown that PHB activity is inhibited by a group of phytochemicals called rocaglamides, which are derived from the traditional Chinese medicinal plants *Aglaia* [39, 56–58]. We therefore investigated whether Roc-A could interfere with EV71 infection cycle by blocking PHB activity in NSC-34 cells. Incubation of Roc-A with virus prior to NSC-34 cell infection (co-treatment) did not result in any significant reduction in viral titer (S4a Fig). When cells were pre-treated with Roc-A prior to EV71 infection (pre-treatment), less than 1 log PFU/mL of decrease in viral titer was observed at the highest drug concentration only (500 nM) (S4b Fig). In contrast, a dose-dependent decrease in the viral titer was seen when Roc-A treatment was applied after infection (post-treatment) at concentrations ranging between 10–100 nM (Fig 8a). Western blot analysis of the cell lysates further confirmed the dose-dependent reduction of intracellular viral capsid protein and PHB (Fig 8b). Taken together, the data suggest that the antiviral effect of Roc-A on EV71-infected NSC-34 cells targets the viral replication step and not the entry step, in contrast to a previous study with HCV [39].

Prior studies focusing on cancer have reported that the mechanism by which Roc-A targets and inhibits PHB activity involves blocking the CRAF/MEK/ERK pathway [56,57], and this was also described in the HCV study [39]. To investigate whether the CRAF/MEK/ERK signaling pathway was inhibited in Roc-A treated NSC-34 cells, Western blot analysis was performed. The results showed that in Roc-A-treated NSC-34 cells only CRAF phosphorylation was impaired while the activation of MEK remained at basal level (S5 Fig). In addition, ERK phosphorylation was not observed under any conditions (S5 Fig). These findings thus indicate that the antiviral effect of Roc-A in NSC-34 cells is likely independent on the CRAF/MEK/ERK signaling pathway.

To decipher the mode of action of Roc-A in NSC-34 cells, immunostaining of Roc-A treated NSC-34 cells was performed. Decreased signals for PHB and mitochondria were observed with increasing concentrations of Roc-A (Fig 8c), suggesting that Roc-A might affect mitochondrial integrity. We thus assessed the mitotoxicity and cytotoxicity of Roc-A using the Mitochondrial ToxGlo assay (Promega). While cytotoxicity remained generally minimal over the range of Roc-A concentrations tested, the intracellular ATP levels were significantly reduced in a dose-dependent manner, thus indicating functional impairment of the mitochondria in Roc-A-treated NSC-34 cells (Fig 8d). Consistently, using the membrane-permeant JC-1 dye as an indicator of mitochondrial membrane potential, Roc-A-treated NSC-34 cells displayed marked and dose-dependent mitochondrial depolarization as evidenced by the decrease of red fluorescent J-aggregates, compared to untreated cells (S6 Fig). Together, these observations suggest that Roc-A treatment in NSC-34 cells results in reduced levels of PHB, leading to mitochondrial destabilization and lower ATP production. One could thus speculate that the lack of intact mitochondria and reduced intracellular ATP levels might eventually impact negatively on EV71 replication efficacy.

### Involvement of PHB in EV71 infection cycle is specific to neuronal cells

**Fig 9:**
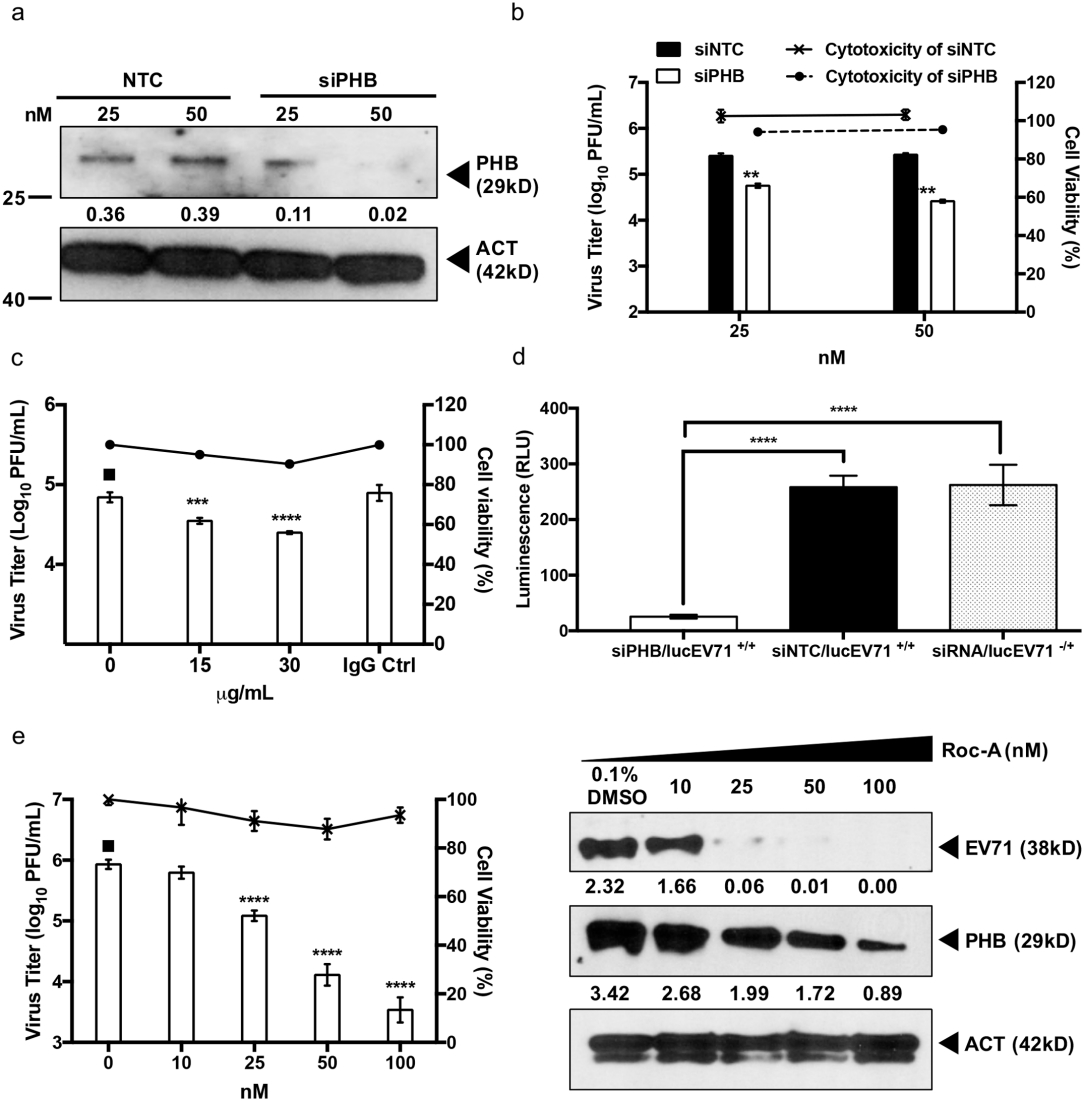
Role of PHB in EV71-infected SK-N-SH cells. (a) SK-N-SH cells were transfected with PHB siRNA at various concentrations for 48 hours. The efficiency of siRNA knockdown was verified by Western blot. PHB-knockdown SK-N-SH cells were infected with EV71 at M.O.I. 1. Viral titers in the culture supernatant were determined by plaque assay at 48 h.p.i. Statistical analysis was performed using two-tailed student’s t-test (** *p*<0.005, *** *p*<0.005). Non-targeting siRNA (NTC) served as control. (b) SK-N-SH cells were pre-treated with anti-PHB antibodies followed by EV71 infection at M.O.I. 1. Culture supernatant was harvested at 48 h.p.i. for viral titer determination by plaque assay. IgG isotype antibodies served as control. Statistical analysis was performed using one-way ANOVA with Dunnett’s post-test (***, *p*<0.0005; ****, *p*<0.0001). (c) SK-N-SH cells were reverse-transfected with 50 nM PHB siRNA, siNTC or left untreated prior to transfection with 0.25 μg of lucEV71 RNA. Luminescence signal was read at 48 h.p.t. Statistical analysis was performed using one-way ANOVA with Dunnet’s post test (**, *p*<0.005). (d) SK-N-SH cells were first infected with EV71 at M.O.I. 1 followed by Roc-A treatment for 48 hours. At 48 h.p.i. the culture supernatant was collected for viral titer determination by plaque assay, and the cell lysate was harvested for Western blot analysis. Statistical analysis was performed using one-way ANOVA with Dunnett’s post-test (**, *p*<0.005). Relative band quantification (below Western blot) was determined by ImageJ, by normalizing to loading control, β-actin. Error bars represent mean ± standard deviation. Cellular cytotoxicity was assessed using alamarBlue assay. One representative from two independent experiments is shown.

The role of PHB in EV71 infection cycle was also studied in human muscle (RD) and neuronal (SK-N-SH) cell lines. As human (GI246483) and murine (GI6679299) PHB display high similarity in their amino acid composition, most of the anti-PHB antibodies commercially available demonstrate good cross reactivity with cell lines of both species. We first showed by flow cytometry comparable levels of surface expression of PHB on RD, SK-N-SH and NSC-34 cells (S7 Fig). However, both PHB gene silencing and PHB receptor blocking experiments performed in human muscle RD cells did not impact the viral titer (S8a and S8b Fig). On the contrary, PHB silencing in the human neuroblastoma cells SK-N-SH led to a significant dose-dependent reduction in viral titer in the culture supernatant (Fig 9a). In addition, reduced virus titers were observed with SK-N-SH cells pre-treated with anti-PHB antibodies, thus supporting that cell surface-expressed PHB is involved in EV71 entry into this human neuroblastoma cell line (Fig 9b). To investigate if intracellular PHB is also involved in viral replication in SK-N-SH cells, transfection of lucEV71 replicon into PHB-silenced SK-N-SH cells was performed. Results showed a significant reduction in the luminescence signal compared to controls (Fig 9c). Finally, the effectiveness of Roc-A treatment in EV71-infected SK-N-SH cells was also assessed. Similar to our observations with NSC-34 cells, a significant dose-dependent decline in viral titers was observed (Fig 9d).

Taken together, these findings thus strongly indicate the specific involvement of PHB in both viral entry and replication of EV71 in neuronal cells from both human and murine origins.

### Roc-A treatment delays development of neurological symptoms and reduces virus titers in the CNS from EV71-infected mice

**Fig 10:**
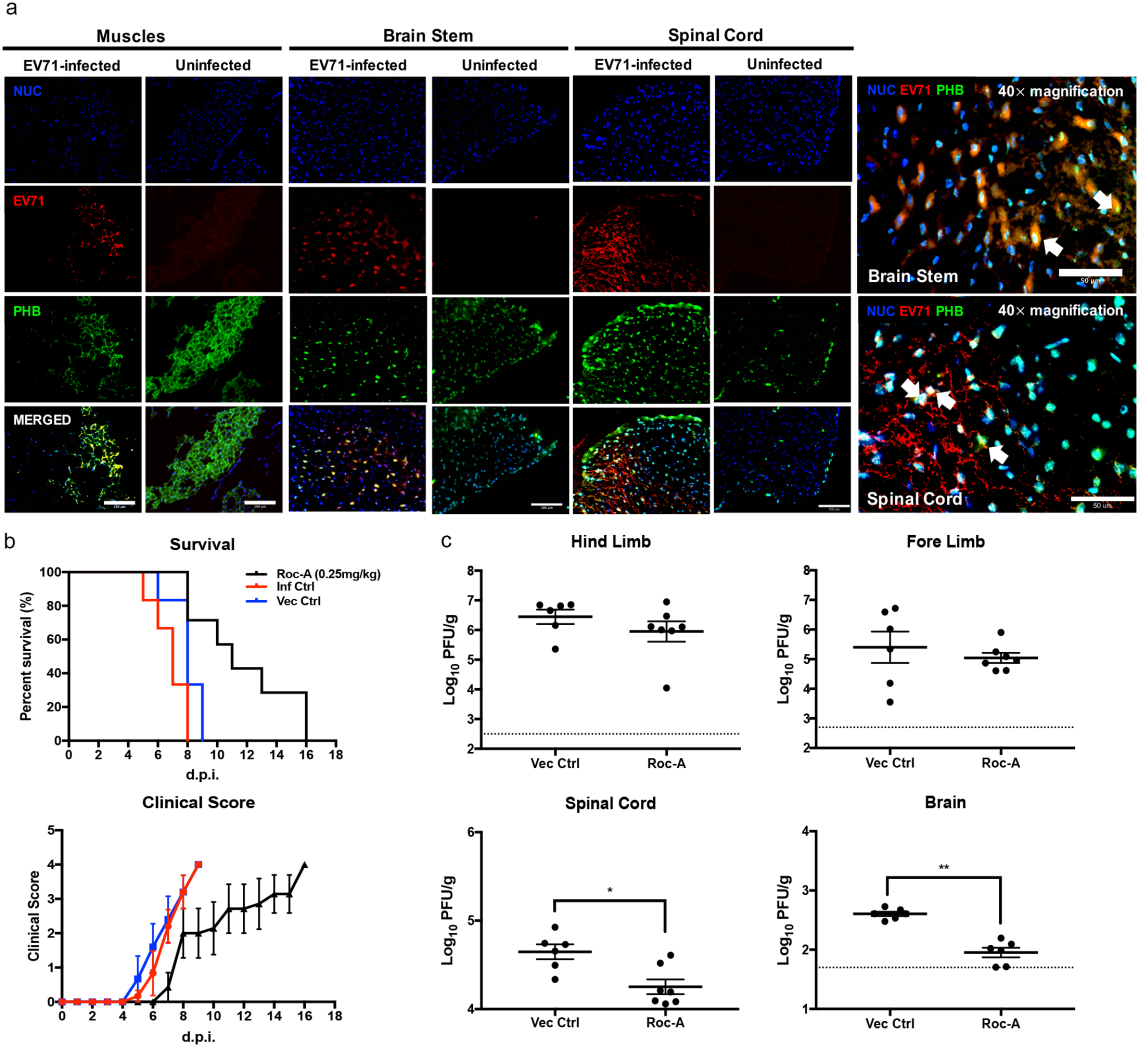
PHB expression and Roc-A treatment in EV71-infected AG129 mice. (a) Two week-old AG129 mice (n = 3) were infected i.p. with EV71 (10^7^ PFU) and the limb muscles, spinal cord and brainstem were harvested for immunohistochemical analysis at day 4 p.i. Scale bars denote 100 μm (20× magnification) and 50 μm (40× magnification). (b) EV71-infected AG129 mice (n = 8) were treated i.p. with Roc-A at 0.25 mg/kg at day 1 and 3 p.i. and were monitored for survival and clinical manifestations. Clinical scores were defined as follows: 0, healthy; 1, ruffled hair and hunched back; 2, limb weakness; 3, one limb paralysis; 4, both limbs paralysis at which point the animals were euthanized. Statistical analysis of survival curve was performed using logrank (Mantel-Cox) test (**, *p*<0.005). (c) Limb muscles, spinal cord and brain were harvested at day 4 p.i. for viral load determination by plaque assay (n = 6/7). Dotted line represents the limit of detection. Statistical analysis was performed using Mann-Whitney U test (*, *p*<0.05). Error bars represent mean ± SEM. One representative of two biological repeats is shown.

The role of PHB was further investigated *in vivo*, using the mouse model of EV71 infection that we established previously where 2-week old AG129 mice (deficient in Type I&II IFN pathways) infected with EV71 display progressive limb paralysis and spatio-temporal virus accumulation in the limb muscles, spinal cord and brainstem [59]. Here, immunohistochemical analysis showed that PHB was readily detected in the limb muscles, brainstem and spinal cord at day 4 p.i. (Fig 10a). Furthermore, some co-localization with EV71 was observed (Fig 10a).

Next, the *in vivo* anti-EV71 efficacy of Roc-A was assessed by treating therapeutically EV71-infected mice with Roc-A at day 1 and 3 p.i. The development of clinical manifestations was clearly delayed in the Roc-A-treated mouse group which resulted in increased survival time compared to the untreated or vehicle-treated control groups (Fig 10b). In addition, the viral loads in limb muscles, spinal cord and brain in both Roc-A-treated and vehicle-treated mice were determined. Comparable viral loads were detected in the limb muscles from both groups (Fig 10c). In contrast, viral titers in the spinal cord and brain from the Roc-A-treated animals were significantly lower compared to the vehicle-treated group (Fig 10c), thus supporting that Roc-A treatment specifically impairs EV71 neuropathogenesis. These findings correlate well with our *in vitro* data showing that the role of PHB during EV71 infection cycle is specific to neuronal cells.

Together, the *in vivo* data support that PHB plays a critical role in EV71 neurovirulence, and that Roc-A represents a potential therapeutic strategy to limit EV71 neuropathogenesis, thereby minimizing neurological manifestations and complications.

## Discussion

The re-emergence of neurotropic enteroviruses in recent years has motivated investigations into EV71 transmission in the neuronal system. Understanding the interplay between virus and host proteins is likely to result in the identification of potential novel drug targets and development of novel antiviral strategies.

Here, using a proteomics approach, we have identified a panel of host factors that displayed dynamic regulation during the course of EV71 infection in the motor neuron NSC-34 cells. The host protein candidates are mainly involved in cytoskeletal structure maintenance, RNA processing and mitochondrial biogenesis. By employing a siRNA gene silencing approach, we have shown that some of these host factors either facilitate or limit EV71 productive infection. Among these host factors, PHB was found to exert a pro-viral effect as evidenced by the reduced viral titers measured in the culture supernatant of NSC-34 cells when the expression of PHB was down-regulated, and by an increased viral titer in PHB over-expressing cells. PHB is mainly localized on plasma membrane, mitochondria and nucleus, and has been involved in multiple signaling pathways regulated by growth factors, immune response, mitochondrial biogenesis, cell migration, proliferation and survival [50,58]. Interestingly, knockdown in NSC-34 cells of STOML2, which was shown to interact with PHB and participate to mitochondria biogenesis [60], resulted in significant reduction of viral titer, similar to that seen with PHB-knocked down cells. This further supports the involvement of mitochondrial proteins during EV71 infection cycle in NSC-34 cells.

In addition, previous studies have reported the association of PHB with internalization of several viruses, including HCV [39], CHIKV [40], DENV [38], and coronavirus (SARS-CoV) [61]. Furthermore, PHB was shown to promote HIV replication by interacting with the HIV-1 glycoprotein [62].

Using various experimental approaches, we have demonstrated that cell surface-expressed PHB is physically associated with EV71 and is involved in the entry of the virus into NSC-34 cells. On the other hand, by employing a lucEV71 replicon, we have shown that intracellular (mitochondrial) PHB plays a role in EV71 replication activity. This was further supported by the observation that mitochondrial PHB co-localizes with the replicating viral genome (dsRNA) and the non-structural proteins 3D polymerase and 3CD complex. Co-immunoprecipitation and TEM approaches also confirmed the physical proximity and likely interactions between viral complexes/viral particles and mitochondria. These data thus led us to propose that mitochondria could serve as replication site for EV71 in NSC-34 cells. The association of EV71 with mitochondria was reported in a previous study where it was proposed that EV71 could potentially interact with some mitochondrial signaling proteins to evade host anti-viral innate immunity [55].

Previous studies have reported that membrane-bound PHB binds to RAS in a GTP-dependent manner, which in turn activates CRAF kinase and eventually triggers the MAPK pathway [58]. Similar to other flavaglines, Rocaglamide (Roc-A) is a natural product that displays insecticidal, anti-fungal, anti-inflammatory and anti-cancer activities [63]. Mechanistically, Roc-A was found to inhibit CRAF-PHB interactions in tumor cells [64–66], and in an *in vitro* model of HCV infection [39]. In EV71-infected NSC-34 cells, incubation with nM concentrations of Roc-A resulted in a dose-dependent reduction in virus titers. However, the mechanism by which Roc-A exerts its antiviral effect against EV71 in NSC-34 cells does not seem to be mediated by blocking the CRAF/MEK/ERK pathway, given that phosphorylated ERK proteins could not be detected in uninfected NSC-34 cells, suggesting that this pathway is not functional in these cells. Instead, we found that Roc-A-treated cells displayed reduced expression of mitochondrial PHB and lower levels of intracellular ATP, which suggests that mitochondria integrity/functionality is impaired in Roc-A treated NSC-34 cells. Since we showed that mitochondrial PHB is involved in EV71 replication and that mitochondria serve as replication site for this virus, the impact of Roc-A on PHB expression and mitochondria integrity could represent the basis of its antiviral activity. Further investigation is necessary to decipher the molecular mechanisms by which Roc-A affects the expression of mitochondrial PHB.

Interestingly, we found that the role of PHB in EV71 entry and replication was limited to cells of neuronal origin, thus supporting a role of PHB specifically in EV71 neuropathogenesis. This neuro-specific phenotype was also observed *in vivo* where Roc-A-treatment resulted in reduced virus loads in the CNS (spinal cord and brain) only but not in the limb muscles from infected mice, although PHB was readily detected in the muscle cells as well. The cell type-dependent involvement of PHB during EV71 infection likely reflects differential intracellular events with different host factors being engaged during EV71 intracellular life cycle. Since EV71 is known to be able to use multiple receptors to enter host cells, one could speculate that the host factors that are engaged during EV71 infection depend on the entry receptor that is being used by the virus. Further study is necessary to explore this idea.

In conclusion, our work has uncovered a novel host factor that is specifically involved in EV71 neurovirulence. In addition, our data support that Roc-A, a previously established anti-cancer drug that targets PHB, could represent a therapeutic approach to limit EV71 neuropathogenesis, and thus prevent or limit associated neurological complications. Given the current attrition in effective antiviral drugs against EV71, Roc-A repurposing is worth considering seriously.

## Materials and methods

### Ethics statement

All the animal experiments were carried out under the guidelines of the National Advisory Committee for Laboratory Animal Research (NACLAR) in the AAALAC-accredited NUS animal facilities. The animal experiments described in this work were approved under the NUS Institutional Animal Care and Use Committee (IACUC) protocol number 16–0136. Non-terminal procedures were performed under anesthesia, and all efforts were made to minimize suffering.

### Cells and virus

Murine motor neuron NSC-34 cells (CELLutions Biosystems, CLU140), human rhabdomyosarcoma (RD) cells (ATCC CCL-136) and human neuroblastoma SK-N-SH cells (ATCC HTB-11) were used in this study. All cell lines were cultured in Dulbecco’s Modified Eagle’s medium (DMEM) (Gibco) containing 10% fetal bovine serum (FBS) (Gibco) at 37°C with 5% CO_2_. Non-mouse-adapted EV71 S41 (5865/SIN/00009, Accession No.: AF316321), kindly provided by Prof. Chow V. T. K. at National University of Singapore, was isolated from the lymph node of a EV71-infected patient who died of encephalitis and pulmonary edema in Singapore [67]. The virus stocks were made in RD cells and the viral titers were determined by plaque assay on RD cells.

### EV71 infection of cell lines

Generally, viral infection was performed at a multiplicity of infection (M.O.I.) of 10 (NSC-34 cells) or 1 (SK-N-SH cells) for 48 hours, prior to downstream viral titer determination or cell lysate harvesting. RD cells (M.O.I. 1) were infected with EV71 for 12 hours before the culture supernatant or cell lysate was collected for further processing.

### Proteomic analysis of EV71-infected NSC-34 cells

NSC-34 cells (10^7^ cells/flask) were seeded onto T-75 culture flask overnight prior to infection at MO.I. 10. After 1 hour incubation, unbound viruses were removed by washes and fresh DMEM with 2% FBS was added to the cells. At 6, 24, 48 and 72 hour post-infection (h.p.i.), the culture medium was removed and cells were washed twice with wash buffer (ProteoExtract Complete Mammalian Proteome Extraction Kit, Millipore). The cells were then gently scrapped off using cell scrapper in 1 mL of wash buffer and spun down at 150 ×g for 10 minutes at 4°C. The cell pellet was stored at -80°C until further processing.

Total proteins extract from infected cells was prepared using ProteoExtract Complete Mammalian Proteome Extraction Kit (Millipore). Briefly, the cell pellet was thawed by resuspension in ice-cold Resuspension Buffer and the proteins were extracted with Extraction Buffer at room temperature (RT). Benzonase and reducing agent were added during protein extraction to minimize nucleic acid contamination and to remove disulphide bonds, respectively. The solubilized protein suspension was subjected to centrifugation at 25,000 ×g for 30 minutes at 4°C to remove the remaining insoluble material. The recovered cell extract was stored at -20°C until further analysis.

The protein samples (200 μg, quantified using RC DC Bradford assay, BioRad) were loaded onto individual lanes of the isoelectronic focusing (IEF) tray with pre-wetted electrode wicks. Passive rehydration was performed for each protein sample using 11cm pH4-7 ReadyStrip IPG strips (BioRad) for 12 hours at RT with gel side down configuration. After rehydration, the protein sample was subjected to IEF on Protean IEF Cell i11 (BioRad) according to the following conditions: 250 V for 20 minutes with linear ramp, 8,000 V for 2.5 hours with linear ramp and 8,000 V for 30,000 V-hours with rapid ramping. IPG strips equilibration was achieved by incubating the strips with pre-warmed DTT Equilibration Buffer I (BioRad) followed by iodoacetamide-supplemented Equilibration Buffer II (BioRad) for 10 minutes each on orbital shaker. Equilibrated IPG strips were then transferred onto 12.5% Tris-HCl Criterion gel (BioRad) and overlaid with ReadyPrep Overlay Agarose (BioRad). Electrophoresis was run at 200 V for 65 minutes.

After electrophoresis, the gels were stained with InstantBlue (Expedeon) for 1 hour and submerged in MilliQ water overnight to remove background signal. Gels were scanned using GS-800 Calibrated Densitometer (BioRad). Gel images were further processed using PDQuest 2-D Analysis Software (BioRad), whereby the different gel images from three independent experiments were matched and the intensities of detected spots were measured. Protein spots that showed at least 0.5-fold change in spot intensity (*p*<0.05, two-tailed Student’s *t*-test), compared to the uninfected control sample, were excised for MALDI-TOF MS. The fold change was calculated using the equation: $\left(\frac{Mean\;of\;Spot\;Expression\;in\;Infected\;Samples}{Mean\;of\;Spot\;Expression\;in\;Uninfected\;Samples}\right)$ for each time point.

### In-gel digestion and mass spectrometry analysis

In-gel digestion and MALDI-TOF MS of the excised protein spots were done by Protein and Proteomics Centre, National University of Singapore (Singapore). The data was search against the murine and viruses National Centre for Biotechnology Information non-redundant (NCBInr) database using a MASCOT program (http://www.matrixscience.com). No threshold was applied to the MS/MS fragment ions intensities.

### Bioinformatics analysis

Data mining of the identified proteins was done by searching in PANTHER (http://www.pantherdb.org/) and Swiss-Prot/TrEMBL (http://www.uniprot.org/) databases. The enrichment analysis of protein-protein interactions was performed using STRING network analysis version 10 (http://string-db.org/). Hierarchical clustering and classification were performed using MultiExperiment Viewer version 4.9 (http://mev.tm4.org/#/welcome).

### Small interfering RNA (siRNA)-mediated gene silencing

On-TARGET plus siRNA SMARTpools targeting genes encoding for prohibitin (PHB), peripherin (PRPH), phosphatidylethanolamine binding protein 1 (PEBP1), enolase-1 (ENO1), stomatin-like protein 2 (STOML2), protein disulfide isomerase family A member 3 (PDIA3), DEP domain containing MTOR-interacting protein (DEPTOR) and non-targeting siRNA control (NTC) were purchased from Dharmacon (GE Life Sciences). The siRNA SMARTpool sequences are shown in S3 Table. Briefly, various concentrations of siRNAs were prepared using DharmaFECT Cell Culture Reagent in a total volume of 50 μL (DCCR) (Dharmacon, GE Life Sciences) and incubated for 5 minutes at RT. DharmaFECT 1 Transfection Reagent (1 μL) (Dharmacon, GE Life Sciences) was then added to the siRNA mixture and topped up to final volume of 100 μL with DCCR. After 30 minutes incubation with transfection reagent at RT, NSC-34 cells (2.5 × 10^5^ cells/ 400 μL) were seeded onto 24 wells plate and reverse transfected with the siRNA constructs. At 48 hour post-transfection (h.p.t.), cellular viability was assessed using alamarBlue cytotoxicity assay (Invitrogen) and the cells were subjected to EV71 infection at M.O.I. 10. Culture supernatant and cell lysate were harvested at 48 h.p.i. for viral titer determination and Western blot analysis. Gene silencing of PHB was also performed in RD and SK-N-SH cells following the same procedure.

### Plaque assay

RD cells (10^5^ cells/well) were seeded onto 24 wells plate. Culture supernatant from EV71-infected samples was serially diluted (10-fold) with DMEM containing 2% FBS prior to infection. The cell monolayer was incubated with 100 μL of the diluted viral suspension for 1 hour at 37°C. The cells were then washed twice with PBS and replaced with 1 mL DMEM containing 2% FBS and 1% carboxymethyl cellulose (CMC, Sigma Aldrich). After 3 days incubation at 37°C, the infected monolayers were fixed and stained with 4% paraformaldehyde/ 0.1% crystal violet solution (Sigma Aldrich). The number of plaques was scored visually and viral titers were expressed as plaque-forming units (PFU) per milliliter (PFU/mL).

### Cellular viability assay

Drug treated or siRNA-transfected cells (2.5×10^4^ NSC-34 or 5×10^4^ SK-N-SH cells/well) were washed twice with PBS and 1× alamarBlue reagent (Invitrogen) diluted with DMEM containing 2% FBS was added. After 4 hours incubation at 37°C, the fluorescence signals were determined using microplate reader (Infinite 2000, Tecan) at Ex_570nm_ and Em_585nm_. Percentage of viable cells was calculated using non-treated cells as control.

### Antibody-mediated blocking assay

NSC-34 and SK-N-SH cells (2×10^5^ cells/well) were seeded onto 24-well plate and incubated at 37°C overnight. The cells were pre-treated with anti-PHB antibody (PA5-27329, Invitrogen) at 5, 15 and 30 μg/mL in DMEM and 2% FBS for 1 hour at 37°C. The cell monolayer was then washed twice with PBS and infected with EV71 at M.O.I. 10 (NSC-34) or 1 (SK-N-SH) for 1 hour. Culture supernatant was harvested at 48 h.p.i. for viral titer determination.

### Viral RNA and siRNA sequential transfection

EV71 viral genome was extracted from infected cell culture supernatant using QIAamp Viral RNA Mini Kit (Qiagen), according to the manufacturer’s instructions. Viral RNA was diluted to 0.25 μg with OptiMEM (Invitrogen) in a total volume of 50 μL and incubated for 5 minutes at RT. After incubation, 1 μL of Lipofectamine 2000 (Invitrogen) was added to the RNA mixture and topped up to 100 μL, followed by 30 minutes incubation at RT. NSC-34 cell suspension (10^5^ cells/ 400 μL) was mixed with 100 μL of transfection mixture and added into each well. At 48 h.p.t. the cells were directly transfected with 50 nM PHB siRNA constructs. The culture supernatant was harvested at different time points for viral titer determination.

### Luciferase EV71 replicon and pCMV6-PHB plasmid transfection

LucEV71 replicon or pCMV6-PHB (Origene)-harbouring *E*. *coli* was grown in LB broth supplemented with kanamycin (50 μg/mL). Both plasmids were extracted using QIAprep Spin Miniprep Kit (Qiagen). LucEV71 was linearized with *Mlu*1 restriction enzyme (R0198S, New England Biolabs). The linearized plasmid was then purified using chloroform/phenol/isoamyl (CPI 24:25:1) and chloroform (Sigma Aldrich). Purified linear plasmid (1 μg) was then subjected to *in vitro* transcription using MEGAscript T7 Transcription Kit (AM1334, ThermoScientific), according to the manufacturer’s instructions. The RNA product was further cleaned-up with chloroform/phenol/isoamyl (CPI 24:25:1) and chloroform. NSC-34 and SK-N-SH cells (2.5×10^4^ cells) seeded in a 96-well plate were reverse-transfected with 50nM siPHB, siNTC or left untreated for 48 hours. At 48 h.p.t. 1 μg of lucEV71 RNA was added into each well and incubated for 48 hours. The luminescence signal was then captured using Nano-Glo Luciferase Assay System (Promega, N1103). Signal was normalized against non-transfected cells. The pCMV6-PHB plasmid (0.5 μg) was transfected into NSC-34 cells for 48 hours prior to viral infection. The culture supernatant was harvested at 48 h.p.i. for determination of viral titer by plaque assay.

### Proximity ligation assay

NSC-34 and RD cells (10^5^ cells) were seeded onto a coverslip, prior to cell surface viral adsorption at M.O.I. 20 (NSC-34 cells) and 5 (RD cells) for 2 hours at 4°C. The cells were then fixed with 4% PFA for 30 mins at RT. The cells were incubated first with 2% (w/v) BSA in PBS for 1 hour at 37°C and probed with primary antibodies (S4 Table) for 1 hour at 37°C. Next, the cells were incubated with two PLA probes (DUO92101, Sigma Aldrich) for 1 hour at 37°C, prior to ligation for 30 minutes. Signal amplification of the PLA probes was achieved by incubating the cells with polymerase for 100 minutes at 37°C. The coverslips were then mounted on glass slides with Duolink *In Situ* Mounting Medium with DAPI and viewed under Olympus IX81 fluorescence microscope.

### Co-immunoprecipitation (Co-IP)

NSC-34 cells (2×10^7^ cells/flask) were seeded onto T-175 culture flask. For study of the interaction between EV71 and surface-expressed PHB, the cells were incubated with EV71 at M.O.I. 30 for 2 hours at 4°C before they were lysed using iced cold lysis buffer (ThermoScientific). For study of the interaction between intracellular PHB and EV71, infected protein extracts (M.O.I. 10) were prepared using iced cold lysis buffer at 48 h.p.i. After conjugating Dynabeads Protein G (Life Technology) with anti-PHB antibody or IgG isotype control (S5 Table) for 20 mins at RT, the protein complexes were pulled down and subjected to Western blot analysis (S6 Table). Briefly, the protein extracts were incubated with the conjugated magnetic beads and further incubated for 3 hour at 4°C with constant rotating, before eluting using Laemmli buffer. Isotype antibody pull-down and uninfected cells were used as controls.

### Mitochondria isolation

NSC-34 (10^7^ cells/flask) and RD (10^7^ cells/flask) cells were seeded onto T-75 culture flask overnight, prior to EV71 infection at M.O.I. 10 and 1, respectively. At 48 h.p.i. (NSC-34) and 12 h.p.i. (RD), the cells were lysed and the mitochondria-enriched fraction was obtained using Mitochondria Isolation Kit for Cultured Cells (89874, ThermoScientific). Both cell fraction and mitochondrial fraction were stored separately for Western blot analysis using relevant antibodies (S4 Table).

### Rocaglamide (Roc-A) treatment of EV71-infected NSC-34 cells

NSC-34 (2×10^5^ cells/well) and SK-N-SH (10^5^ cells/well) cells were seeded onto 24-well plate and incubated overnight. For pre-treatment condition, the cells were incubated with Roc-A (SML0656, Sigma Aldrich) at various concentrations for 3 hours prior to EV71 infection at M.O.I. 10. For post-treatment condition, the cells were infected with EV71 at M.O.I. 1 (NSC-34 cells) or 1 (SK-N-SH cells) for 1 hour and then treated with Roc-A for 48 hours. For co-treatment, cells were incubated with EV71 and Roc-A simultaneously for 1 hour prior washing and replaced with fresh 2% DMEM. Culture supernatant was harvested at 48 h.p.i. for viral titer determination.

### Mitotoxicity assay

NSC-34 (5×10^4^ cells/well) cells were seeded onto 96-well white opaque plate and incubated overnight. The cells were treated with various concentrations of Roc-A (diluted with 2% DMEM) for 48 hours. Cytotoxicity and mitotoxicity of Roc-A were then assessed using Mitochondrial ToxGlo Assay (G8000, Promega). Briefly, 20 μL of 5× Cytotoxicity reagent were added into each well and incubated at 37°C for 30 minutes. Cytotoxicity was measured using fluorescence at Ex_485nm_ and Em_525nm_. After equilibrating the assay plate to RT for 10 minutes, 100 μL of ATP detection reagent were added into each well and mitotoxicity was measured by luminescence. All readings were normalized against non-treated cells. Sodium azide (S2002, Sigma Aldrich) and staurosporine (S6942, Sigma Aldrich) were used as mitochondrial toxin and cytotoxin positive controls, respectively.

### Mitochondrial membrane potential assay

NSC-34 (10^5^ cells/well) cells were seeded onto 8-wells chamber slides (Ibidi) and incubated overnight. The cells were treated with various concentrations of Roc-A (diluted with 2% DMEM) for 48 hours. JC-1 dye (Invitrogen) (10 μg/mL in 2% DMEM) was added into each well and incubated for another 15 mins, prior to imaging. NSC-34 cells incubated with sodium azide NaN3 at 1 μM in 2% DMEM (S2002, Sigma Aldrich) was used as positive control.

### Western blot analysis

Cell lysate was prepared using M-PER Mammalian Protein Extraction Reagent containing 1% of Halt Protease Inhibitor Cocktail and 1% of 0.5 M EDTA (ThermoScientific). Protein quantification was performed using Quick Start Bradford Protein Assay (BioRad). Denatured proteins (5 μg) were resolved in 10% SDS-PAGE gel and transferred electrophoretically onto nitrocellulose membrane using Trans-Blot Turbo Transfer System (BioRad). After blocking with 5% (w/v) milk in TBST (TBS buffer with 0.01% Tween 20) for 1 hour at RT, the membrane was probed using specific primary antibodies and relevant secondary antibodies (S6 Table). The chemiluminescence signal was visualized using Clarity Western ECL Substrate (BioRad) on X-ray films. Densitometric quantification was performed using ImageJ and the relative band intensity was normalized against β-actin.

### Indirect immunofluorescence assay (IFA)

NSC-34 and RD cells (10^5^ cells) were seeded onto coverslips, incubated overnight and infected with EV71 at M.O.I. 10 and 1, respectively. At 48 h.p.i. (NSC-34) and 12 h.p.i. (RD), the cells were fixed with iced cold methanol for 15 minutes at -20°C. The cells were then washed extensively with PBS to remove residual methanol and blocked with 5% (w/v) Bovine Serum Albumin (BSA) (Sigma Aldrich) in PBS for 1 hour at 37°C, prior to immunostaining with relevant antibodies (S7 Table). The nucleus was revealed using NucBlue Live ReadyProbes Reagent (Molecular Probes). Fluorescence images were captured using Olympus IX81 microscope and further processed using ImageJ.

Limb muscles, spinal cord and brainstem from EV71-infected AG129 mice were harvested at day 4 p.i. after systemic perfusion. The organs were fixed in 4% PFA overnight prior immersing in 15% and 30% sucrose solution, and embedded in Tissue-Tek OCT (VWR) solution. The organ samples were then frozen at -80°, sectioned (10 μm) using a cryostat (Leica) and mounted on a glass slide prior to blocking and staining as described above.

### Transmission electron microscopy (TEM)

Samples were fixed for 1h at RT with 2.5% glutaraldehyde containing 1% Tannic acid in 0.1M cacodylate buffer (pH 7.2), then washed three times for 5 min (each time) in 0.1M cacodylate buffer and post-fixed for 1h at RT with 1% osmium tetroxide in the same buffer. Samples were then dehydrated in a graded series of ethanol and embedded in Spurr. Thin sections were stained with 2% uranyl acetate and lead citrate and observations were performed by transmission electron microscopy using a FEI Tecnai Spirit G2 at 100Kv. Image were taken using a FEI Eagle 4K CCD camera.

### Flow cytometry

NSC-34, SK-N-SH (8 × 10^6^ cells) and RD (4 × 10^6^ cells) cells were seeded onto 6-well plate overnight. The cells were dislodged by incubating them in 10 mM EDTA/PBS in 4°C for 10 mins and spun down to collect the cell pellet. Cells were then blocked with either human or murine Fc-blocker (564200 or 553142, BD Pharmigen; 1:400) for 30 min at RT, and subsequently stained with anti-PHB antibody (AB75766, Abcam; 1:200) and/or anti-rabbit AF488 antibody (A27034, ThermoScientific; 1:500) for 30 min at 4°C. Stained cells were then fixed with 4% PFA. Flow cytometry analysis was carried out using the Becton-Dickinson Fortessa flow cytometer and analysed using FlowJo v10.

### Roc-A treatment of EV71-infected AG129 mice

Two-week old AG129 mice (B&K Universal) were bred and housed under specific pathogen-free conditions in individual ventilated cages. Infection was performed by injecting intraperitoneally (i.p.) 10^7^ PFU of S41 (in 100 μL) per mouse. At day 1 and 3 post-infection (p.i.), mice were injected i.p. with 0.25 mg/kg of Roc-A (in 0.25% DMSO in sterile olive oil). The control group was inoculated with 0.25% DMSO in olive oil. Clinical manifestations were observed for a period of 20 days. Clinical score was graded as follows: 0, healthy; 1, ruffled hair and hunched back; 2, limb weakness; 3, one limb paralysis; 4, two limbs paralysis; and 5, death. Two limbs paralysis was used as criterion for early euthanasia. For virus titer determination, infected mice were euthanized at day 4 p.i. and perfused with 50 mL of sterile PBS systemically. The fore and hind limb muscles, spinal cord and brain were harvested and weighed before mechanical homogenization in 1 mL of serum-free DMEM. The homogenates were spun down at 10,000 rpm for 10 minutes at 4°C, and clarified using a 0.22 μm filter before serial dilution was carried out for plaque assay. Viral titers were expressed as PFU per gram of tissue.

## Supporting information

S1 Table Spot analysis of EV71-infected NSC-34 cells. A total of 81 spots were excised and identified via MALDI-TOF MS/MS.
(DOCX)

S2 Table Abbreviation of proteins for STRING and PANTHER enrichment analysis. (DOCX)

S3 Table Sequences of On-TARGET plus siRNA SMARTpools. (DOCX)

S4 Table Antibodies used in proximity ligation assay. (DOCX)

S5 Table Antibodies used in co-immunoprecipitation experiments. (DOCX)

S6 Table Primary and secondary antibodies used for Western blot analysis. (DOCX)

S7 Table Primary and secondary antibodies used in immunofluorescence assay (IFA). (DOCX)

S1 Fig Proteomics data analysis. (a) Enrichment analysis was performed using GO Ontology (PANTHER database) (http://pantherdb.org/). (b) Meta-analysis of EV71 proteomic studies. The number or percentage (in bracket) of candidates proteins identified is indicated.
(PDF)

S2 Fig Modulation of PHB expression in NSC-34 cells during EV71 infection. NSC-34 cells were infected with EV71 at MOI 10. Cell lysates were harvested at indicated time point and subjected to Western blot analysis. Relative band quantification (below Western blot) was determined by ImageJ, by normalizing to loading control, β-actin. Two biological replicates were performed and one representative data was shown.
(PDF)

S3 Fig Effect of down-regulation or over-expression of PHB on EV71 viral output. **(a-c) Down-regulation of PHB.** Individual siRNA was reversed transcribed into NSC-34 cells. At 48 h.p.t., the knockdown efficiency was determined by (a) Western blot and (b) the cell viability was assessed via alamarBlue cytotoxicity assay. (c) PHB-knocked down NSC-34 cells were infected with EV71 at M.O.I. 10 and viral titers in the culture supernatant were determined at 48 h.p.i by plaque assay. Non-targeting siRNA (siNTC) serves as control. Statistical analysis was performed using two-way ANOVA with Dunnett’s post-test (**, *p*<0.005; ***, *p*<0.0005; ****, *p*<0.0001). **(d-e) Over-expression of PHB.** (d) 0.5 μg of pCMV6-PHB was transfected into NSC-34 cells for 48 hours, prior to infection with EV71 at M.O.I. 5. Viral titer was determined at 48 h.p.i. Non-transfected cells (Empty Ctrl) and cells transfected with vector only (Vector Ctrl) served as controls. Relative band quantification (below Western blot) was determined by ImageJ, by normalizing to loading control, β-actin. Statistical analysis was performed using two-tailed student’s t-test (**, *p*<0.005). One representative from two independent experiments is shown.
(PDF)

S4 Fig Roc-A treatment of EV71-infected NSC-34 cells. (a) For co-treatment assay, virus was incubated with Roc-A for 1 hour before adding the mixture onto the cells. After one hour of incubation on cell monolayer, the mixture was then removed and replaced with fresh 2% DMEM. (b) For pre-treatment assay, the cells were pre-treated with Roc-A for 3 hours, prior to viral infection. Culture supernatants were harvested at 48 h.p.i. for viral titer determination by plaque assay. Cell viability was assessed using alamarBlue viability assay. Statistical analysis was performed using one-way ANOVA with Dunnett’s post-test (**, *p*<0.005). One representative from two independent experiments is shown.
(PDF)

S5 Fig Western blot analysis of Roc-A treated NSC-34 cells. EV71-infected or uninfected NSC-34 cells were treated with Roc-A at concentrations of 50 and 100nM. At 48 hours post-incubation, the cell lysates were harvested and subjected to Western blot analysis. 0.1% DMSO-treated, uninfected and infected only cells served as control. UI, uninfected; INF, infected. One representative from two independent experiments is shown.
(PDF)

S6 Fig JC1-mitochondrial membrane potential assay. NSC-34 cells were incubated with various concentrations of Roc-A for 48 hours before assessment of mitochondrial membrane potential using JC-1 dye, with green-fluorescent monomer at depolarized membrane potentials or red-fluorescent J-aggregate at hyperpolarized membrane potentials (healthy mitochondria). Representative images were shown. Scale bar denotes 20 μm.
(PDF)

S7 Fig Detection of PHB at the cell surface by FACS. Overlay histogram showing NSC-34, SK-N-SH and RD cells stained with anti-PHB antibody (red line). The cells were blocked with respective Fc blocker and stained with anti-PHB antibody or secondary antibody, prior to fixing with 4% PFA. Secondary antibody—stained cells (blue line) served as control. One representative of two biological repeats is shown. Median Fluorescent Intensity (MFI) values are shown for each cell line.
(PDF)

S8 Fig (a) siRNA-mediated PHB silencing and (b) PHB receptor blocking in EV71-infected RD cells. (a) RD cells were transfected with PHB siRNA at various concentrations. The efficiency of siRNA knockdown was confirmed by Western blot. PHB-knockdown cells were infected with EV71 S41 at M.O.I. 1. Viral titer in the culture supernatant was determined by plaque assay at 12 h.p.i. Statistical analysis was performed using two-tailed student’s t-test (* *p*<0.05, ** *p*<0.005, *** *p*<0.005, **** *p*<0.0001). Error bars represent mean ± standard deviation. Relative band quantification (below Western blot) was determined by ImageJ, by normalizing to loading control, β-actin. Non-targeting siRNA (NTC) served as control. (b) RD cells were pre-incubated with anti-PHB antibody or isotype control antibody for 1 hour before infection. Culture supernatant was harvested at 12 h.p.i. for viral titer determination. Cell viability was determined using alamarBlue cytotoxicity assay. One representative of two biological repeats is shown.
(PDF)
