# Supplementary material for: Prohibitin plays a critical role in Enterovirus 71 neuropathogenesis
Source: PLoS Pathog. 2018 Jan 11;14(1):e1006778. doi: 10.1371/journal.ppat.1006778 (PMC5764453; doi:10.1371/journal.ppat.1006778)
Supplement: S1 Table — A total of 81 spots were excised and identified via MALDI-TOF MS/MS. (DOCX) [file ppat.1006778.s001.docx]

| **S1 Table. Spot analysis of EV71-infected NSC-34 cells.** A total of 81 spots were excised and identified via MALDI-TOF MS/MS. | | | | | | | | | | | | | |
| --- | --- | --- | --- | --- | --- | --- | --- | --- | --- | --- | --- | --- | --- |
| **Spot** | **6 h.p.i.** | **24 h.p.i.** | | **48 h.p.i.** | **72 h.p.i.** | | **Protein Name** | **Mascot score** | **Peptides identified** | **Sequence coverage (%)** | **Mr (Dalton)** | **Expt. pI** | **NCBI ID** |
| **Apoptosis Regulation** | | | | | | | | | | | | | |
| 14 | 0.477 | 1.318 | | 0.631 | 0.771 | | Gag polyprotein | 271 | 11 | 21 | 60988 | 7.14 | GI\|120864 |
| 67 | 1.060 | 1.675 | | 1.034 | 0.903 | | DEP domain-containing mTOR-interacting protein | 45 | 10 | 22 | 46603 | 8.03 | GI\|145558896 |
| 79 | 2.725 | 1.267 | | 0.478 | 1.053 | | Acidic leucine-rich nuclear phosphoprotein | 77 | 4 | 11 | 28691 | 3.99 | GI\|46395611 |
|  |  |  | |  |  | |  |  |  |  |  |  |  |
| **ATP Synthesis** | | | | | | | | | | | | | |
| 29 | 0.184 | 0.939 | | 1.508 | 1.563 | | ATP synthase subunit d, mitochondrial | 124 | 9 | 49 | 18795 | 5.52 | GI\|25089776 |
| 36 | 0.644 | 0.611 | | 0.943 | 2.050 | | Cytochrome b-c1 complex subunit 1, mitochondrial | 341 | 20 | 31 | 53446 | 5.81 | GI\|341941780 |
| 42 | 0.271 | 0.785 | | 0.864 | 0.894 | | V-type proton ATPase catalytic subunit A | 97 | 19 | 22 | 68625 | 5.42 | GI\|145559539 |
| 55 | 0.345 | 0.716 | | 1.388 | 0.165 | | ADP-sugar pyrophosphatase | 187 | 6 | 16 | 24254 | 5.34 | GI\|44888250 |
|  |  |  | |  |  | |  |  |  |  |  |  |  |
| **Biogenesis** | | | | | | | | | | | | | |
| 12 | 1.279 | 1.693 | | 1.016 | 1.053 | | Stomatin-like protein 2, mitochondrial | 173 | 11 | 26 | 38475 | 8.95 | GI\|60415940 |
| 66 | 0.567 | 1.251 | | 0.917 | 1.239 | | Nucleophosmin | 44 | 4 | 15 | 32711 | 4.62 | GI\|2500582 |
|  |  |  | |  |  | |  |  |  |  |  |  |  |
| **Calcium Binding** | | | | | | | | | | | | | |
| 43 | 0.819 | 1.774 | | 0.530 | 0.683 | | Filaggrin | 25 | 7 | 9 | 35658 | 6.58 | GI\|120164 |
|  |  |  | |  |  | |  |  |  |  |  |  |  |
| **Cell Proliferation Regulator** | | | | | | | | | | | | | |
| 27 | 0.597 | 1.593 | | 2.356 | 1.675 | Prohibitin | | 508 | 17 | 52 | 29859 | 5.57 | GI\|54038837 |
| 62 | 1.486 | 0.262 | | 1.123 | 0.575 | Phosphatidylethanolamine-binding protein 1 | | 264 | 7 | 32 | 20988 | 5.19 | GI\|29840839 |
| 69 | 1.189 | 0.871 | | 1.105 | 0.937 | Proliferating cell nuclear antigen | | 375 | 18 | 49 | 29108 | 4.66 | GI\|129695 |
|  |  |  | |  |  |  | |  |  |  |  |  |  |
| **Cytoskeleton Organization** | | | | | | | | | | | | | |
| 3 | 0.932 | 1.501 | | 0.131 | 0.950 | Fascin | | 568 | 31 | 56 | 55215 | 6.44 | GI\|146345421 |
| 11 | 1.316 | 1.064 | | 0.000 | 1.785 | Actin, cytoplasmic 2 | | 401 | 22 | 53 | 42108 | 5.31 | GI\|54036677 |
| 26 | 0.741 | 3.509 | | 2.439 | 1.669 | F-actin-capping protein subunit beta | | 216 | 14 | 26 | 31611 | 5.47 | GI\|1345668 |
| 32 | 0.479 | 0.302 | | 1.205 | 0.096 | Actin, cytoplasmic 1 | | 230 | 15 | 31 | 42052 | 5.29 | GI\|46397334 |
| 33 | 7.970 | 1.914 | | 2.089 | 0.619 | Actin, cytoplasmic 1 | | 241 | 14 | 31 | 42052 | 5.29 | GI\|46397334 |
| 34 | 1.891 | 1.809 | | 0.491 | 1.042 | Actin, cytoplasmic 1 | | 215 | 15 | 31 | 42052 | 5.29 | GI\|46397334 |
| 37 | 0.566 | 1.167 | | 0.604 | 6.725 | Actin-like protein 6A | | 94 | 12 | 20 | 47931 | 5.39 | GI\|23396474 |
| 39 | 1.257 | 0.643 | | 0.317 | 0.559 | Tubulin alpha-1B chain | | 28 | 6 | 12 | 50804 | 4.94 | GI\|55977764 |
| 46 | 1.542 | 1.188 | | 1.904 | 2.268 | Tubulin alpha-1C chain | | 496 | 27 | 48 | 50562 | 4.96 | GI\|55977482 |
| 47 | 1.035 | 0.970 | | 1.357 | 2.247 | Tubulin beta-4B chain | | 804 | 40 | 67 | 50255 | 4.79 | GI\|55977481 |
| 48 | 1.447 | 1.004 | | 2.065 | 0.422 | Drebrin-like protein | | 213 | 26 | 33 | 48955 | 4.90 | GI\|51315842 |
| 52 | 0.676 | 0.811 | | 1.157 | 1.376 | Actin, cytoplasmic 2 | | 260 | 14 | 31 | 42108 | 5.31 | GI\|54036677 |
| 53 | 1.318 | 1.242 | | 3.077 | 0.306 | Actin, cytoplasmic 2 | | 497 | 20 | 43 | 42108 | 5.31 | GI\|54036677 |
| 54 | 0.717 | 1.708 | | 7.458 | 2.272 | Actin, cytoplasmic 2 | | 591 | 17 | 48 | 42108 | 5.31 | GI\|54036677 |
| 70 | 0.950 | 1.118 | | 1.172 | 1.902 | Keratin, type II cytoskeletal 75 | | 61 | 11 | 14 | 59932 | 8.46 | GI\|81896062 |
| 74 | 0.790 | 1.143 | | 0.700 | 0.977 | Translationally-controlled tumor protein | | 97 | 4 | 23 | 19564 | 4.76 | GI\|51703328 |
| 76 | 0.393 | 1.132 | | 1.287 | 1.412 | Myosin-4 | | 40 | 20 | 11 | 223632 | 5.58 | GI\|73921192 |
| 78 | 0.894 | 1.027 | | 0.941 | 1.021 | Myosin-6 | | 39 | 20 | 11 | 224225 | 5.57 | GI\|3024204 |
|  |  |  | |  |  |  | |  |  |  |  |  |  |
| **Homeostasis** | | | | | | | | | | | | | |
| 35 | 0.389 | 0.838 | 0.608 | | 0.699 | Glutaredoxin-3 | | 87 | 5 | 13 | 38039 | 5.42 | GI\|37089726 |
| 60 | 0.521 | 2.077 | 0.786 | | 0.778 | Rho GDP-dissociation inhibitor | | 95 | 9 | 33 | 23450 | 5.12 | GI\|21759130 |
|  |  |  |  | |  |  | |  |  |  |  |  |  |
| **Metabolism** | | | | | | | | | | | | | |
| 2 | 0.973 | 0.520 | 0.940 | | 1.151 | Aldehyde dehydrogenase, mitochondrial | | 594 | 27 | 46 | 57015 | 7.53 | GI\|1352250 |
| 5 | 0.771 | 2.170 | 1.286 | | 1.112 | D-3-phosphoglycerate dehydrogenase | | 678 | 25 | 41 | 57347 | 6.12 | GI\|55584180 |
| 6 | 0.753 | 2.413 | 0.996 | | 1.329 | Glucose-6-phosphate 1-dehydrogenase X | | 781 | 40 | 61 | 59681 | 6.06 | GI\|134047776 |
| 7 | 0.953 | 0.969 | 5.813 | | 1.054 | Alpha-enolase | | 872 | 29 | 59 | 47453 | 6.37 | GI\|13637776 |
| 9 | 1.015 | 0.639 | 0.861 | | 0.952 | Alpha-enolase | | 773 | 27 | 55 | 47453 | 6.37 | GI\|13637776 |
| 13 | 1.393 | 1.847 | 0.739 | | 0.920 | Phosphoglycerate mutase 1 | | 807 | 24 | 60 | 28928 | 6.67 | GI\|20178035 |
| 24 | 3.057 | 3.094 | 1.085 | | 1.024 | Ornithine aminotransferase, mitochondrial | | 292 | 20 | 28 | 48723 | 6.19 | GI\|266683 |
| 28 | 0.553 | 1.304 | 1.164 | | 2.290 | Adenylate kinase isoenzyme 1 | | 106 | 8 | 37 | 21640 | 5.67 | GI\|13959400 |
| 30 | 0.559 | 1.113 | 2.106 | | 2.047 | Pyruvate dehydrogenase E1 component subunit beta, mitochondrial | | 153 | 19 | 37 | 39254 | 6.41 | GI\|46396509 |
| 50 | 1.640 | 1.190 | 0.164 | | 1.392 | Inorganic pyrophosphatase | | 228 | 17 | 45 | 33102 | 5.37 | GI\|52783095 |
| 59 | 0.262 | 1.133 | 0.505 | | 0.848 | Cysteine sulfinic acid decarboxylase | | 37 | 9 | 17 | 55737 | 6.17 | GI\|27151478 |
| 65 | 1.633 | 1.335 | 1.031 | | 1.104 | Sulfotransferase 6B1 | | 53 | 13 | 30 | 35397 | 5.47 | GI\|269969671 |
|  |  |  |  | |  |  | |  |  |  |  |  |  |
| **Molecular chaperone** | | | | | | | | | | | | | |
| 1 | 1.253 | 1.714 | 0.173 | | 1.137 | T-complex protein 1 subunit gamma | | 223 | 4 | 8 | 61162 | 6.28 | GI\|549059 |
| 8 | 0.823 | 4.407 | 0.755 | | 1.452 | T-complex protein 1 subunit beta | | 937 | 35 | 64 | 57783 | 5.97 | GI\|22654291 |
| 23 | 0.939 | 1.888 | 1.558 | | 2.441 | T-complex protein 1 subunit epsilon | | 347 | 27 | 37 | 60042 | 5.72 | GI\|549058 |
| 40 | 0.722 | 0.847 | 2.156 | | 1.510 | 60 kDa heat shock protein, mitochondrial | | 789 | 22 | 32 | 61088 | 5.91 | GI\|51702252 |
| 41 | 0.000 | 0.804 | N.D | | N.D | 60 kDa heat shock protein, mitochondrial | | 102 | 11 | 21 | 61088 | 5.91 | GI\|51702252 |
| 44 | 1.176 | 0.990 | 1.819 | | 0.566 | 60 kDa heat shock protein, mitochondrial | | 210 | 16 | 24 | 61088 | 5.91 | GI\|51702252 |
| 49 | 2.010 | 1.159 | 1.060 | | 1.172 | Endoplasmin | | 456 | 42 | 38 | 92703 | 4.74 | GI\|119362 |
|  |  |  |  | |  |  | |  |  |  |  |  |  |
| **Neuronal Intermediate Filament** | | | | | | | | | | | | | |
| 38 | 0.512 | 0.369 | 2.109 | | 0.258 | Peripherin | | 167 | 13 | 22 | 54349 | 5.40 | GI\|3334475 |
|  |  |  |  | |  |  | |  |  |  |  |  |  |
| **Nucleic Acid Synthesis** | | | | | | | | | | | | | |
| 10 | 1.386 | 0.795 | 0.382 | | 0.620 | TAR DNA-binding protein 43 | | 871 | 24 | 46 | 44918 | 6.26 | GI\|20140642 |
| 18 | 0.906 | 0.413 | 0.594 | | 0.256 | 40S ribosomal protein S8 | | 336 | 13 | 48 | 24475 | 10.3 | GI\|54039529 |
| 21 | 5.309 | 2.947 | 0.635 | | 1.434 | Adenine phosphoribosyltransferase | | 102 | 11 | 43 | 19883 | 6.31 | GI\|449081285 |
| 68 | 0.134 | 1.133 | 0.708 | | 0.561 | Histone-lysine N-methyltransferase setd3 | | 43 | 13 | 14 | 67533 | 5.47 | GI\|81879567 |
| 80 | 1.614 | 0.716 | 0.553 | | 1.418 | Ribonuclease inhibitor | | 254 | 11 | 19 | 51495 | 4.69 | GI\|78099143 |
|  |  |  |  | |  |  | |  |  |  |  |  |  |
| **Protein Modification** | | | | | | | | | | | | | |
| 4 | 3.094 | 0.945 | 0.698 | | 0.000 | Pre-mRNA-processing factor 19 | | 391 | 18 | 45 | 55661 | 6.14 | GI\|55976574 |
| 15 | 1.138 | 1.629 | 0.762 | | 1.410 | Eukaryotic translation initiation factor 4H | | 446 | 18 | 40 | 27381 | 6.67 | GI\|15214055 |
| 17 | 0.770 | 2.116 | 0.812 | | 0.609 | Proteasome subunit alpha type-1 | | 447 | 18 | 55 | 29813 | 6.00 | GI\|9910833 |
| 19 | 6.750 | 1.850 | 1.705 | | 0.903 | Proteasome subunit beta type-7 | | 153 | 14 | 50 | 30214 | 8.14 | GI\|17380252 |
| 20 | 0.087 | 0.757 | 0.385 | | 0.202 | Proteasome subunit beta type-3 | | 384 | 22 | 51 | 23235 | 6.15 | GI\|9910831 |
| 22 | 2.241 | 1.456 | 1.128 | | 1.451 | Protein disulfide-isomerase A3 | | 114 | 19 | 30 | 57099 | 5.88 | GI\|146345480 |
| 25 | 0.115 | 0.494 | N.D | | 1.580 | 60S acidic ribosomal protein P0 | | 27 | 10 | 24 | 34366 | 5.91 | GI\|46397819 |
| 31 | 0.447 | 1.247 | 1.309 | | 1.417 | Eukaryotic translation initiation factor 3 subunit I | | 165 | 15 | 24 | 36837 | 5.38 | GI\|20138778 |
| 45 | 2.239 | 1.095 | 1.365 | | 0.325 | Heterogeneous nuclear ribonucleoprotein K | | 253 | 21 | 32 | 51230 | 5.39 | GI\|48429104 |
| 51 | 0.733 | 3.193 | 1.568 | | 1.486 | Eukaryotic translation initiation factor 3 subunit F | | 210 | 15 | 31 | 38074 | 5.33 | GI\|341940488 |
| 56 | 0.433 | 0.663 | 0.513 | | 3.490 | Serine/arginine-rich splicing factor 1 | | 523 | 21 | 52 | 27842 | 10.3 | GI\|68725069 |
| 57 | 2.647 | 0.984 | 1.530 | | 0.516 | Proteasome subunit alpha type-3 | | 196 | 8 | 16 | 28615 | 5.29 | GI\|3914438 |
| 61 | 0.843 | 1.266 | 0.739 | | 0.835 | Ubiquitin carboxyl-terminal hydrolase isozyme L1 | | 76 | 9 | 46 | 25165 | 5.14 | GI\|18203410 |
| 64 | 0.954 | 0.863 | 0.753 | | 1.332 | 40S ribosomal protein SA | | 657 | 21 | 52 | 32931 | 4.80 | GI\|146345507 |
| 73 | 1.379 | 0.693 | 1.253 | | 1.226 | Proteasome subunit alpha type-5 | | 474 | 14 | 56 | 26565 | 4.74 | GI\|12229953 |
| 75 | 0.894 | 0.996 | 0.069 | | 0.718 | S-phase kinase-associated protein 1 | | 50 | 2 | 7 | 18831 | 4.40 | GI\|54036535 |
|  |  |  |  | |  |  | |  |  |  |  |  |  |
| **Redox Regulation** | | | | | | | | | | | | | |
| 16 | 2.304 | 1.687 | 0.607 | | 0.739 | Peroxiredoxin-6 | | 910 | 30 | 84 | 24969 | 5.71 | GI\|3219774 |
| 58 | 2.114 | 1.074 | 1.663 | | 0.604 | Chloride intracellular channel protein 1 | | 594 | 15 | 47 | 27338 | 5.09 | GI\|6685328 |
| 63 | 0.958 | 1.358 | 0.920 | | 0.577 | Peroxiredoxin-2 | | 601 | 17 | 55 | 21936 | 5.20 | GI\|2499469 |
| 81 | 0.955 | 0.689 | 1.297 | | 1.888 | Chloride intracellular channel protein 1 | | 594 | 15 | 47 | 27338 | 5.09 | GI\|6685328 |
|  |  |  |  | |  |  | |  |  |  |  |  |  |
| **Signalling Pathway** | | | | | | | | | | | | | |
| 71 | 1.527 | 0.984 | 1.501 | | 2.130 | 14-3-3 protein gamma | | 428 | 21 | 59 | 28456 | 4.80 | GI\|48428722 |
| 72 | 1.190 | 1.119 | 0.543 | | 3.179 | 14-3-3 protein zeta/delta | | 692 | 21 | 60 | 27925 | 4.73 | GI\|52000885 |
| 77 | 1.348 | 0.600 | 0.590 | | 1.528 | 14-3-3 protein epsilon | | 551 | 20 | 47 | 29326 | 4.63 | GI\|60391192 |
| ^**N.D = Not detected^ | | | | | | | | | | | | | |
