# Supplementary material for: Prohibitin plays a critical role in Enterovirus 71 neuropathogenesis
Source: PLoS Pathog. 2018 Jan 11;14(1):e1006778. doi: 10.1371/journal.ppat.1006778 (PMC5764453; doi:10.1371/journal.ppat.1006778)
Supplement: S2 Table — (DOCX) [file ppat.1006778.s002.docx]

| **S2 Table. Abbreviation of proteins for STRING and PANTHER enrichment analysis.** | |
| --- | --- |
| **Protein name** | **Abbreviation** |
| T-complex protein 1 subunit gamma | CCT3 |
| Aldehyde dehydrogenase, mitochondrial | ALDH2 |
| Fascin | FSCN1 |
| Pre-mRNA-processing factor 19 | PRPF19; CWC15; BCAS2 |
| D-3-phosphoglycerate dehydrogenase | 3-PGDH |
| Glucose-6-phosphate 1-dehydrogenase X | G6PD1 |
| Alpha-enolase | ENO1 |
| T-complex protein 1 subunit beta | CCT2 |
| Alpha-enolase | ENO1 |
| TAR DNA-binding protein 43 | TARDBP |
| Actin, cytoplasmic 2 | ACTG |
| Stomatin-like protein 2, mitochondrial | STOML2 |
| Phosphoglycerate mutase 1 | PGAM1 |
| Gag polyprotein | GAG |
| Eukaryotic translation initiation factor 4H | EIF4H |
| Peroxiredoxin-6 | PRDX6 |
| Proteasome subunit alpha type-1 | PSMA1 |
| 40S ribosomal protein S8 | RPS8 |
| Proteasome subunit beta type-7 | PSMB7 |
| Proteasome subunit beta type-3 | PSMB3 |
| Adenine phosphoribosyltransferase | APRT |
| Protein disulfide-isomerase A3 | PDIA3 |
| T-complex protein 1 subunit epsilon | TCPE |
| Ornithine aminotransferase, mitochondrial | OAT |
| 60S acidic ribosomal protein P0 | RPLP0 |
| F-actin-capping protein subunit beta | CAPZB |
| Prohibitin | PHB |
| Adenylate kinase isoenzyme 1 | AK1 |
| ATP synthase subunit d, mitochondrial | ATP5H |
| Pyruvate dehydrogenase E1 component subunit beta, mitochondrial | PDHA1 |
| Eukaryotic translation initiation factor 3 subunit I | EIF3I |
| Actin, cytoplasmic 1 | ACTB |
| Glutaredoxin-3 | GLRX3 |
| Cytochrome b-c1 complex subunit 1, mitochondrial | UQCRFS1 |
| Actin-like protein 6A | ACTL6A |
| Peripherin | PRPH |
| Tubulin alpha-1B chain | TUBA1B |
| 60 kDa heat shock protein, mitochondrial | HSPD1 |
| V-type proton ATPase catalytic subunit A | ATP6V1A |
| Filaggrin | FLG |
| Heterogeneous nuclear ribonucleoprotein K | HNRPK |
| Tubulin alpha-1C chain | TUBA1C |
| Tubulin beta-4B chain | TUBB4B |
| Drebrin-like protein | DBNL |
| Endoplasmin | HSP90B1 |
| Inorganic pyrophosphatase | PPA1 |
| Eukaryotic translation initiation factor 3 subunit F | EIF3F |
| Actin, cytoplasmic 2 | ACTG |
| ADP-sugar pyrophosphatase | NUDT5 |
| Serine/arginine-rich splicing factor 1 | SRSF1 |
| Proteasome subunit alpha type-3 | PSMA3 |
| Chloride intracellular channel protein 1 | CLIC1 |
| Cysteine sulfinic acid decarboxylase | CSAD |
| Rho GDP-dissociation inhibitor | ARHGDIA |
| Ubiquitin carboxyl-terminal hydrolase isozyme L1 | UCHL1 |
| Phosphatidylethanolamine-binding protein 1 | PEBP1 |
| Peroxiredoxin-2 | PRDX2 |
| 40S ribosomal protein SA | RPSA |
| Sulfotransferase 6B1 | ST6B1 |
| Nucleophosmin | NPM |
| DEP domain-containing mTOR-interacting protein | DEPTOR |
| Histone-lysine N-methyltransferase setd3 | SETD3 |
| Proliferating cell nuclear antigen | PCNA |
| Keratin, type II cytoskeletal 75 | KRT75 |
| 14-3-3 protein gamma | YWHAG |
| 14-3-3 protein zeta/delta | YWHAZ |
| Proteasome subunit alpha type-5 | PSMB5 |
| Translationally-controlled tumor protein | TCTP |
| S-phase kinase-associated protein 1 | SKP1 |
| Myosin-4 | MYH4 |
| 14-3-3 protein epsilon | YWHAE |
| Myosin-6 | MYH6 |
| Acidic leucine-rich nuclear phosphoprotein | ANP32A |
| Ribonuclease inhibitor | RNH1 |
| Chloride intracellular channel protein 1 | CLIC1 |
