# Supplementary material for: Prohibitin plays a critical role in Enterovirus 71 neuropathogenesis
Source: PLoS Pathog. 2018 Jan 11;14(1):e1006778. doi: 10.1371/journal.ppat.1006778 (PMC5764453; doi:10.1371/journal.ppat.1006778)
Supplement: S3 Table — (DOCX) [file ppat.1006778.s003.docx]

| **S3 Table. Sequences of On-TARGET plus siRNA SMARTpools.** | | |
| --- | --- | --- |
| **Gene** | **NCBI Gene ID** | **Target Sequence** |
| **Prohibitin (PHB_9,10,11,12)** | 18673 | UCAAUAUCACACUGCGAAU,  CCGAGGACAUUGCGUACCA,  CCUCCAUUCUGCCGUAUAU,  GGAUGGACUUGGACGGGAC |
| **Peripherin (PRPH)** | 19132 | GCCUAGAACUGGAGCGCAA.  ACGAGAACAUCGCGGCAAA,  CCUCGGAGCGCCUCGAUUU,  AGGCAGAGGAGUGGUAUAA |
| **Phosphatidylethanolamine binding protein 1 (PEBP1)** | 23980 | GCUUGCAGGAGGUGGACGA,  CAGAUCAGGUGGCUGCGUA,  GCACAGGUCUCCACCGCUA,  GGAUAGAGGUUUAGGGUGU |
| **Enolase-1 (ENO1)** | 13806 | GCAUUGGAGCAGAGGUUUA,  AGUGAAUGUUGUGGAGCAA,  UGACCAACCCUAAGCGGAU,  GCUCAAGACUGCAAUCGCA |
| **Stomatin-like protein 2 (STOML2)** | 66592 | CGUCUUUGGCCGUCCACUU,  UAAAGUGACCAGUGGCAAA,  GGUGUGAGGAGUUGAUUCU,  GUGCAGAGUCUCAAGGAAA |
| **Protein disulfide isomerase family A member 3 (PDIA3)** | 14827 | GUAUGAAGGUGGCCGUGAA,  CAUAUGAAGUCAAGGGUUU,  CCUCAUAUGACGGAAGAUA,  CUUACUAUGAUGUGGACUA |
| **DEP domain containing MTOR-interacting protein (DEPTOR)** | 97998 | ACCGAGAGACGGCGAUAAA,  GGGUGAAGUAUGAGCGGAC,  GCAAGGAAGACAUUCACGA,  ACACAACAGUGGCGAGGCA |
