# Supplementary material for: Prohibitin plays a critical role in Enterovirus 71 neuropathogenesis
Source: PLoS Pathog. 2018 Jan 11;14(1):e1006778. doi: 10.1371/journal.ppat.1006778 (PMC5764453; doi:10.1371/journal.ppat.1006778)
Supplement: S4 Table — (DOCX) [file ppat.1006778.s004.docx]

| **S4 Table. Antibodies used in Proximity Ligation Assay.** | | | |
| --- | --- | --- | --- |
|  | **Primary Antibody** | | |
|  | **PHB** | **SCARB-2** | **EV71** |
| **NSC-34 cells** | Anti-PHB antibody (1:200, PA527329, Invitrogen) |  | Anti-EV71 antibody (1:1000, MAB979, Millipore) |
|  |  | Anti-SCARB-2 antibody (1:50, PA520540, Invitrogen) | Anti-EV71 antibody (1:1000, MAB979, Millipore) |
| **RD cells** |  | Anti-SCARB-2 antibody (1:50, PA520540, Invitrogen) | Anti-EV71 antibody (1:1000, MAB979, Millipore) |
