# Supplementary material for: Prohibitin plays a critical role in Enterovirus 71 neuropathogenesis
Source: PLoS Pathog. 2018 Jan 11;14(1):e1006778. doi: 10.1371/journal.ppat.1006778 (PMC5764453; doi:10.1371/journal.ppat.1006778)
Supplement: S5 Table — (DOCX) [file ppat.1006778.s005.docx]

| **S5 Table. Antibodies used in co-immunoprecipitation experiments.** | | |
| --- | --- | --- |
|  | **co-IP Antibody** | **Western Blot Antibody** |
| **EV71 Infection** | Anti-PHB antibody (16 μg, PA527329, Invitrogen, 0.8mg/mL) | Anti-EV71 antibody (10 µg, MAB979, Millipore) |
|  |  | Anti-EV71 3D antibody (1:1000, GTX630193, Genetex) |
| **Mock Infection** | Anti-PHB antibody (16 µg, PA527329, Invitrogen) | Mouse anti-EV71 antibody (1:1000, MAB979, Millipore)  Anti-EV71 3D antibody (1:1000, GTX630193, Genetex) |
| **Isotype Control** | IgG isotype control | Mouse anti-EV71 antibody (1:1000, MAB979, Millipore)  Anti-EV71 3D antibody (1:1000, GTX630193, Genetex) |
