# Supplementary material for: Prohibitin plays a critical role in Enterovirus 71 neuropathogenesis
Source: PLoS Pathog. 2018 Jan 11;14(1):e1006778. doi: 10.1371/journal.ppat.1006778 (PMC5764453; doi:10.1371/journal.ppat.1006778)
Supplement: S6 Table — (DOCX) [file ppat.1006778.s006.docx]

| **S6 Table.** **Primary and secondary antibodies used for Western blot analysis.** | | |
| --- | --- | --- |
|  | **Primary Antibody** | **Secondary Antibody** |
| **Mitochondria** | Anti-ATPB antibody (1:1000, ab14730, Abcam) | HRP-conjugated anti-mouse IgG antibody (1:5000, #172-1011, BioRad) |
| **Prohibitin** | Anti-PHB antibody (1:1000, PA5-27329, Invitrogen) | HRP-conjugated anti-rabbit IgG antibody (1:5000, #170-6515, BioRad) |
| **EV71** | Anti-EV71 antibody (1:1000, MAB979, Millipore) | HRP-conjugated anti-mouse IgG antibody (1:5000, #172-1011, BioRad) |
| **Endoplasmic Reticulum** | Anti-calreticulin antibody (1:5000, ab92516, Abcam) | HRP-conjugated anti-rabbit IgG antibody (1:5000, #170-6515, BioRad) |
| **EV71 3D** | Anti-EV71 3D antibody (1:5000, GTX630193, Genetex) | HRP-conjugated anti-mouse IgG antibody (1:5000, #172-1011, BioRad) |
| **Craf** | Anti-Craf antibody (1:1000, PA5-20970, Invitrogen) | HRP-conjugated anti-chicken IgY antibody (1:250, SA1-300, Invitrogen) |
| **Phosphorylated Craf** | Anti-phospho-c-Raf pSer338 antibody (1:1000, MA5-15176, Invitrogen) | HRP-conjugated anti-rabbit IgG antibody (1:5000, #170-6515, BioRad) |
| **MEK** | Anti-MEK1 antibody (1:500, 13-3500, Invitrogen) | HRP-conjugated anti-mouse IgG antibody (1:5000, #172-1011, BioRad) |
| **Phosphorylated MEK** | Anti-phospho-MEK1 pSer298 antibody (1:500, 44-460G, Invitrogen) | HRP-conjugated anti-rabbit IgG antibody (1:5000, #170-6515, BioRad) |
| **ERK 1/2** | Anti-p44/42 MAPK (Erk1/2) antibody (1:1000, #9102, CST) | HRP-conjugated anti-rabbit IgG antibody (1:5000, #170-6515, BioRad) |
| **Phosphorylated ERK 1/2** | Anti-phospho-p44 MAPK + p42 MAPK pThr202 + pTyr204 antibody (1:1000, 36-8800, Invitrogen) | HRP-conjugated anti-rabbit IgG antibody (1:5000, #170-6515, BioRad) |
| **LC3B** | Anti-LC3B antibody (1:3000, ab51520, Abcam) | Anti-rabbit HRP antibody (1:5000, #170-6515, BioRad) |
| **Actin** | Anti-beta actin antibody (1:20000, ab8227, Abcam) | HRP-conjugated anti-rabbit IgG antibody (1:5000, #170-6515, BioRad) |
