# Supplementary material for: Prohibitin plays a critical role in Enterovirus 71 neuropathogenesis
Source: PLoS Pathog. 2018 Jan 11;14(1):e1006778. doi: 10.1371/journal.ppat.1006778 (PMC5764453; doi:10.1371/journal.ppat.1006778)
Supplement: S7 Table — (DOCX) [file ppat.1006778.s007.docx]

| **S7 Table. Primary and secondary antibodies used in immunofluorescence assay (IFA).** | | |
| --- | --- | --- |
|  | **Primary Antibody** | **Secondary Antibody** |
| **Mitochondria** | Anti-ATP Synthase Subunit IF1 antibody (1:3000, A21355, Invitrogen) | Alexa Fluor 488-conjugated anti-mouse IgG antibody (1:500, A-11001, Invitrogen) |
|  | Alexa Fluor 488-conjugated anti-ATPB (1:100, ab197904, Abcam) | |
| **Prohibitin** | Anti-PHB antibody (1:300, PA5-27329, Invitrogen) | Alexa Fluor 594-conjugated anti-rabbit IgG antibody (1:500, A-21244, Invitrogen) |
|  | Alexa Fluor 488-conjugated anti-PHB (1:50, ab184813, Abcam) | |
| **dsRNA** | Anti-dsRNA antibody (1:300, J2-1513, Eng Scicons) | Alexa Fluor 350-conjugated anti-mouse IgG antibody (1:50, A-11045, Invitrogen)  Alexa Fluor 488-conjugated anti-mouse IgG antibody (1:500, A-11001, Invitrogen) |
| **EV71** | Anti-EV71 antibody (1:1000, MAB979, Millipore) | Alexa Fluor 488-conjugated anti-mouse IgG antibody (1:500, A-11001, Invitrogen) |
|  | Anti-EV71 antibody (1:1000, PA5-32202, Invitrogen) | Alexa Fluor 594-conjugated anti-rabbit IgG antibody (1:500, A-21244, Invitrogen) |
