# Supplementary figures and images for: Prohibitin plays a critical role in Enterovirus 71 neuropathogenesis
Source: PLoS Pathog. 2018 Jan 11;14(1):e1006778. doi: 10.1371/journal.ppat.1006778 (PMC5764453; doi:10.1371/journal.ppat.1006778)

a

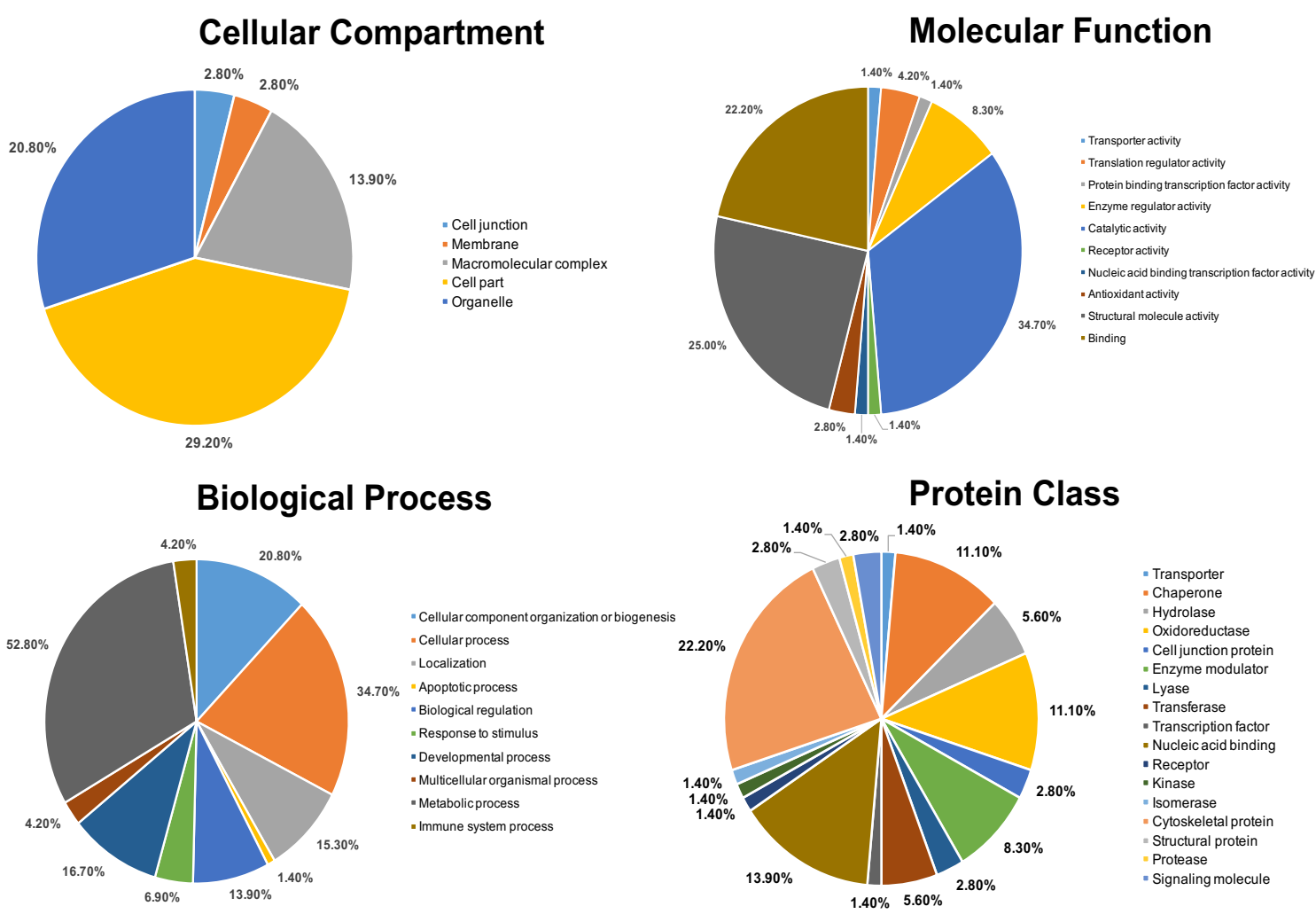

b

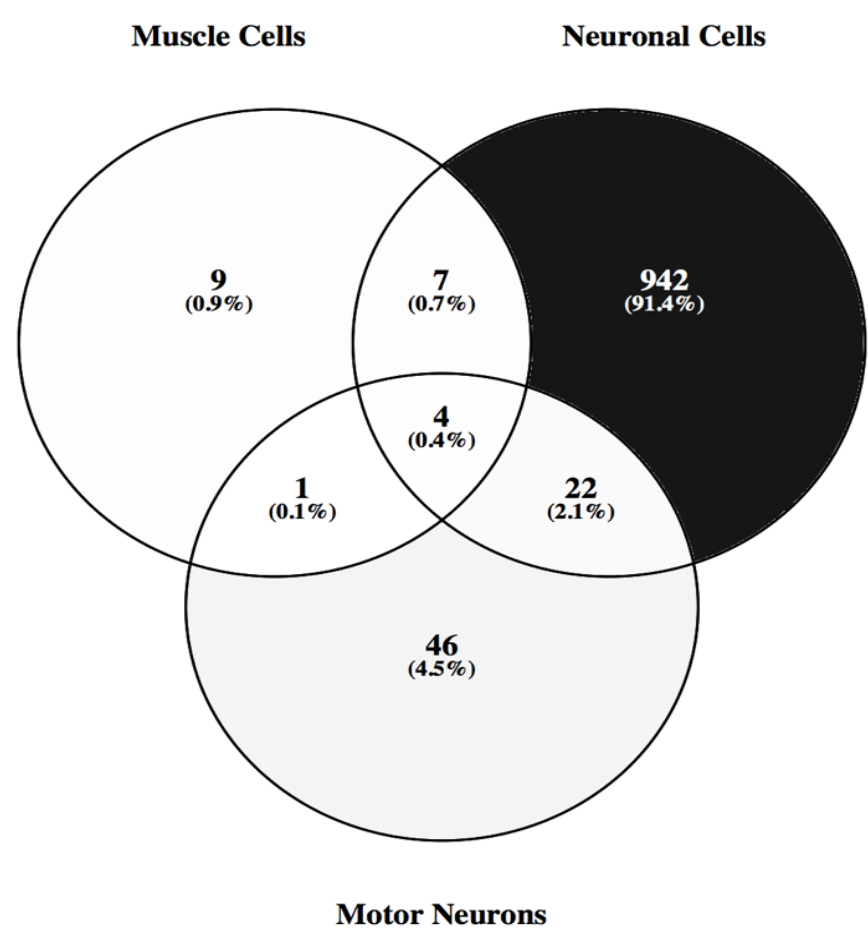

Supplement: S1 Fig — (a) Enrichment analysis was performed using GO Ontology (PANTHER database) (http://pantherdb.org/). (b) Meta-analysis of EV71 proteomic studies. The number or percentage (in bracket) of candidates proteins identified is indicated. (PDF) [file ppat.1006778.s008.pdf]

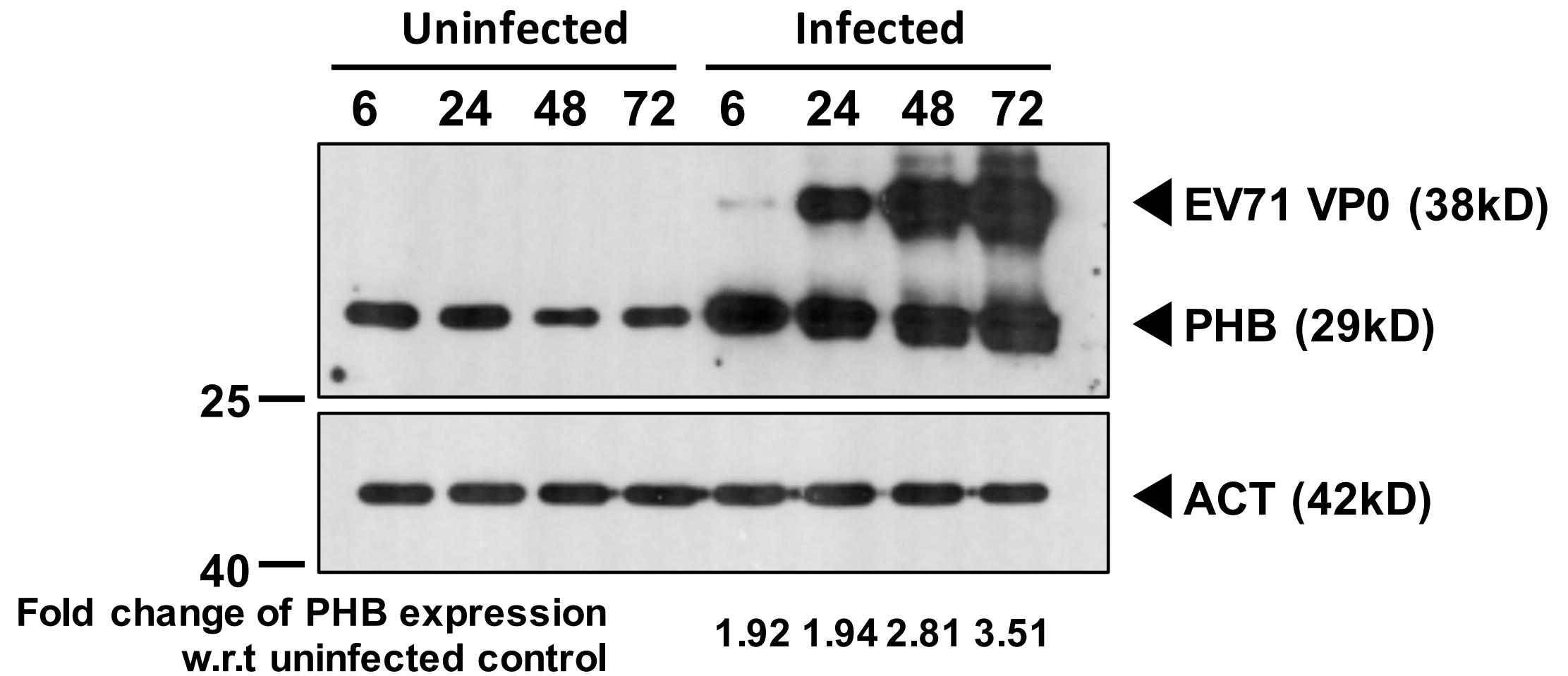

Supplement: S2 Fig — NSC-34 cells were infected with EV71 at MOI 10. Cell lysates were harvested at indicated time point and subjected to Western blot analysis. Relative band quantification (below Western blot) was determined by ImageJ, by normalizing to loading control, β-actin. Two biological replicates were performed and one representative data was shown. (PDF) [file ppat.1006778.s009.pdf]

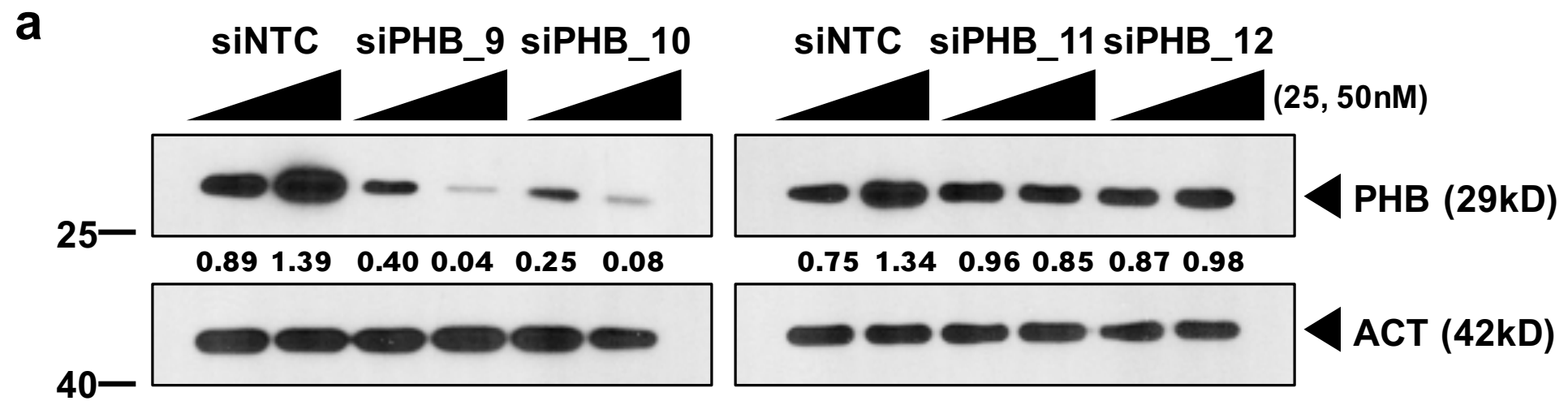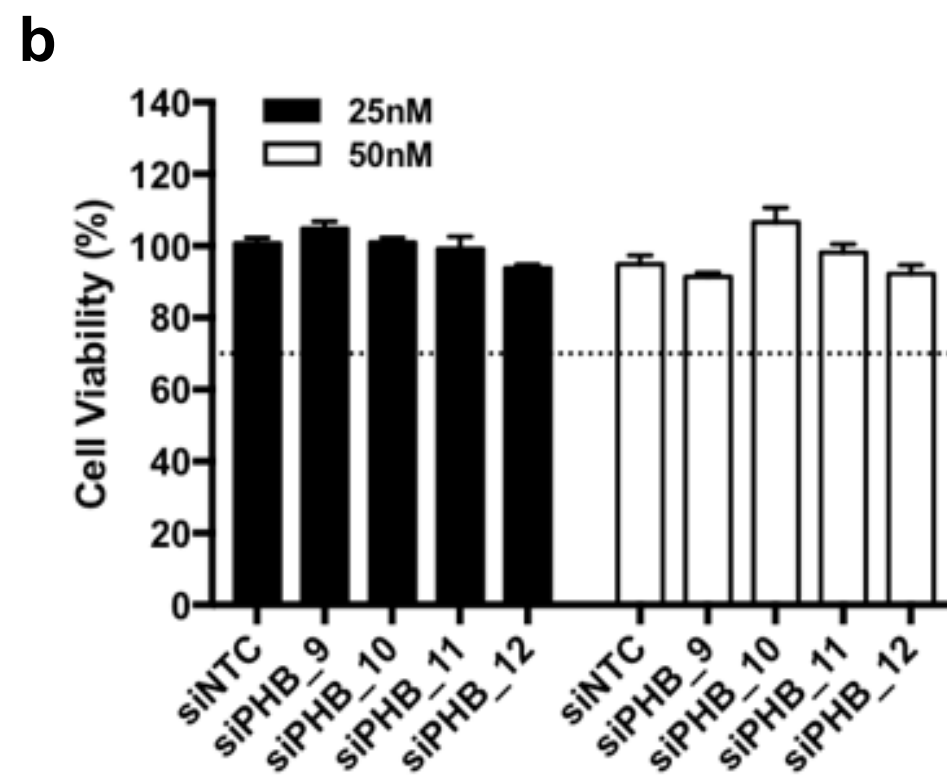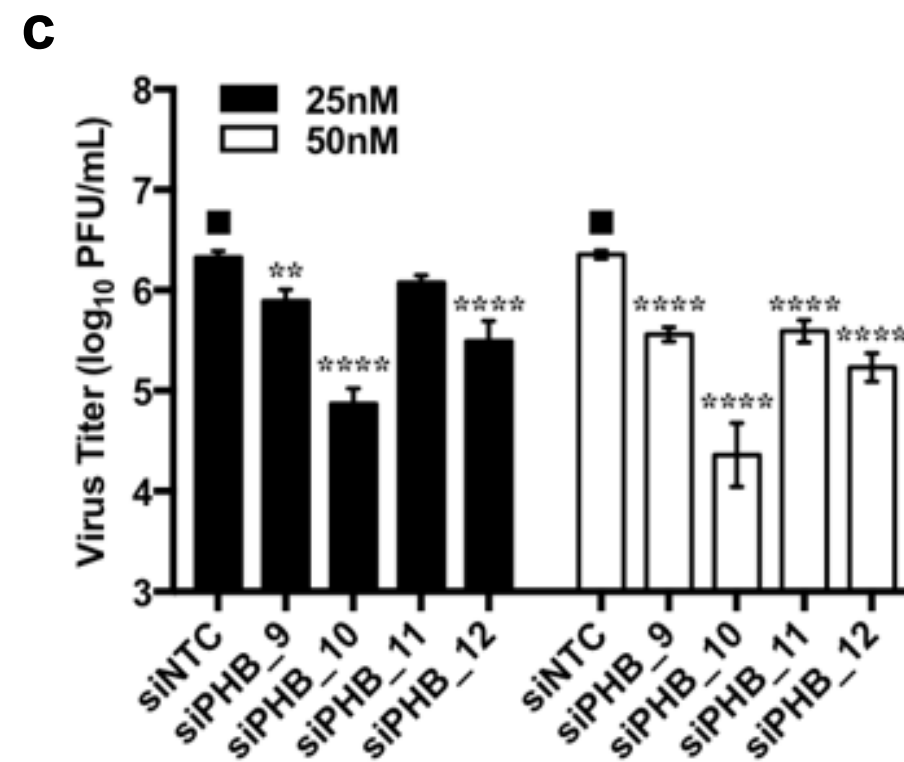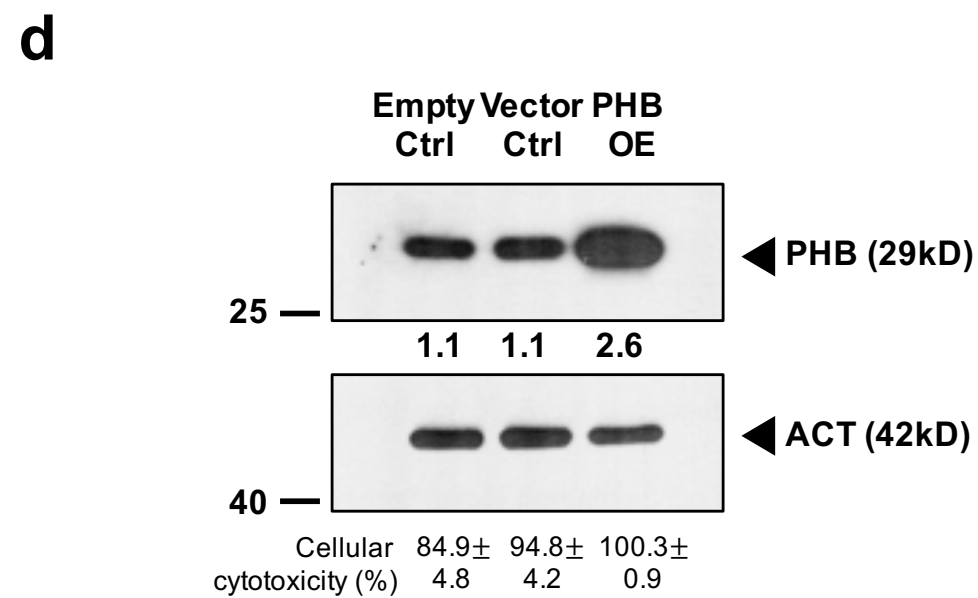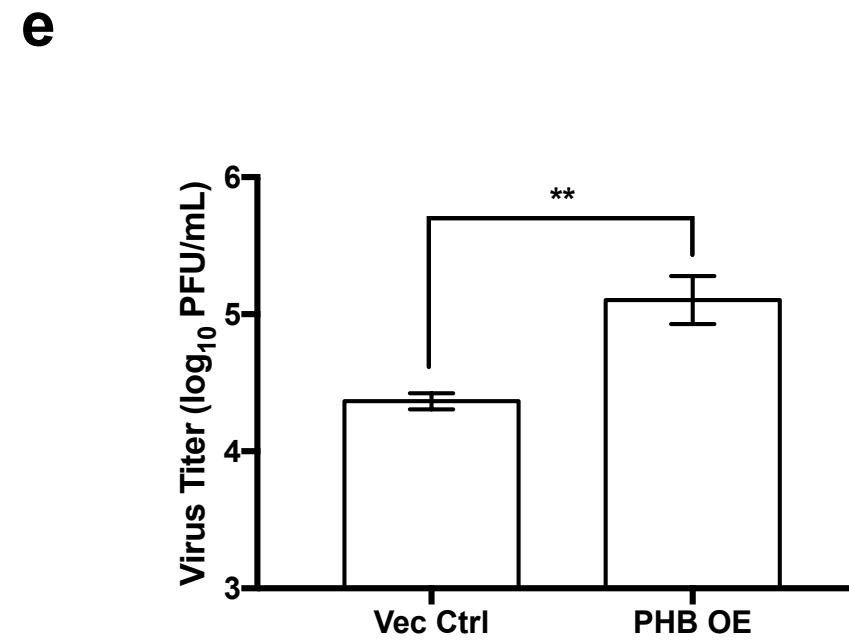

Supplement: S3 Fig — (a-c) Down-regulation of PHB. Individual siRNA was reversed transcribed into NSC-34 cells. At 48 h.p.t., the knockdown efficiency was determined by (a) Western blot and (b) the cell viability was assessed via alamarBlue cytotoxicity assay. (c) PHB-knocked down NSC-34 cells were infected with EV71 at M.O.I. 10 and viral titers in the culture supernatant were determined at 48 h.p.i by plaque assay. Non-targeting siRNA (siNTC) serves as control. Statistical analysis was performed using two-way ANOVA with Dunnett’s post-test (**, p<0.005; ***, p<0.0005; ****, p<0.0001). (d-e) Over-expression of PHB. (d) 0.5 μg of pCMV6-PHB was transfected into NSC-34 cells for 48 hours, prior to infection with EV71 at M.O.I. 5. Viral titer was determined at 48 h.p.i. Non-transfected cells (Empty Ctrl) and cells transfected with vector only (Vector Ctrl) served as controls. Relative band quantification (below Western blot) was determined by ImageJ, by normalizing to loading control, β-actin. Statistical analysis was performed using two-tailed student’s t-test (**, p<0.005). One representative from two independent experiments is shown. (PDF) [file ppat.1006778.s010.pdf]

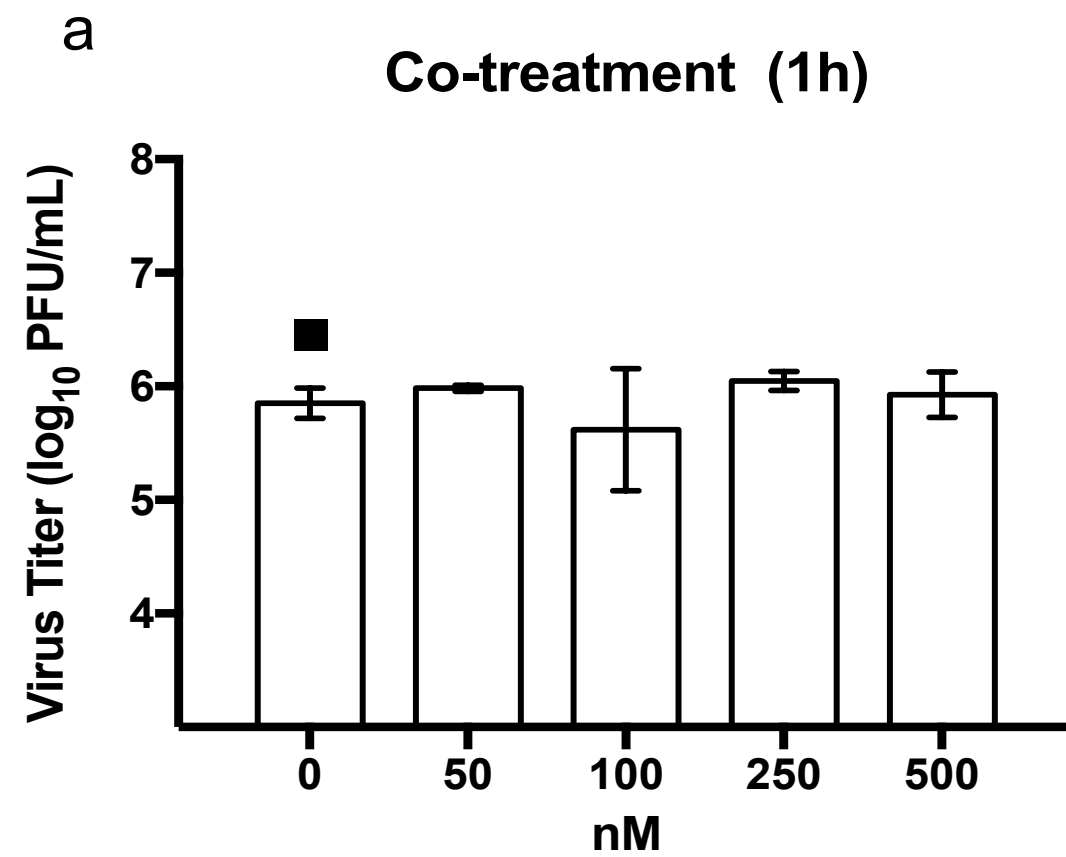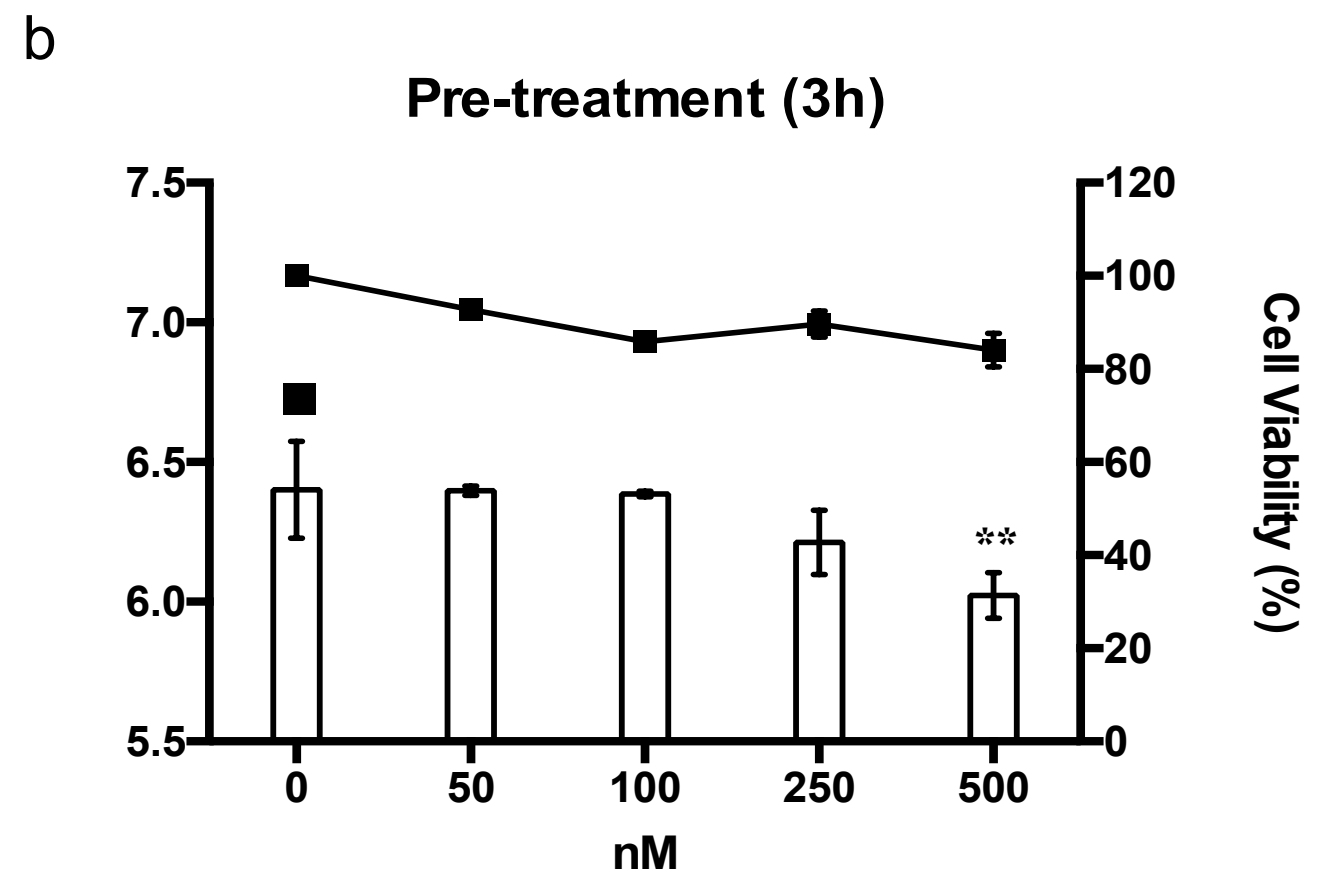

Supplement: S4 Fig — (a) For co-treatment assay, virus was incubated with Roc-A for 1 hour before adding the mixture onto the cells. After one hour of incubation on cell monolayer, the mixture was then removed and replaced with fresh 2% DMEM. (b) For pre-treatment assay, the cells were pre-treated with Roc-A for 3 hours, prior to viral infection. Culture supernatants were harvested at 48 h.p.i. for viral titer determination by plaque assay. Cell viability was assessed using alamarBlue viability assay. Statistical analysis was performed using one-way ANOVA with Dunnett’s post-test (**, p<0.005). One representative from two independent experiments is shown. (PDF) [file ppat.1006778.s011.pdf]

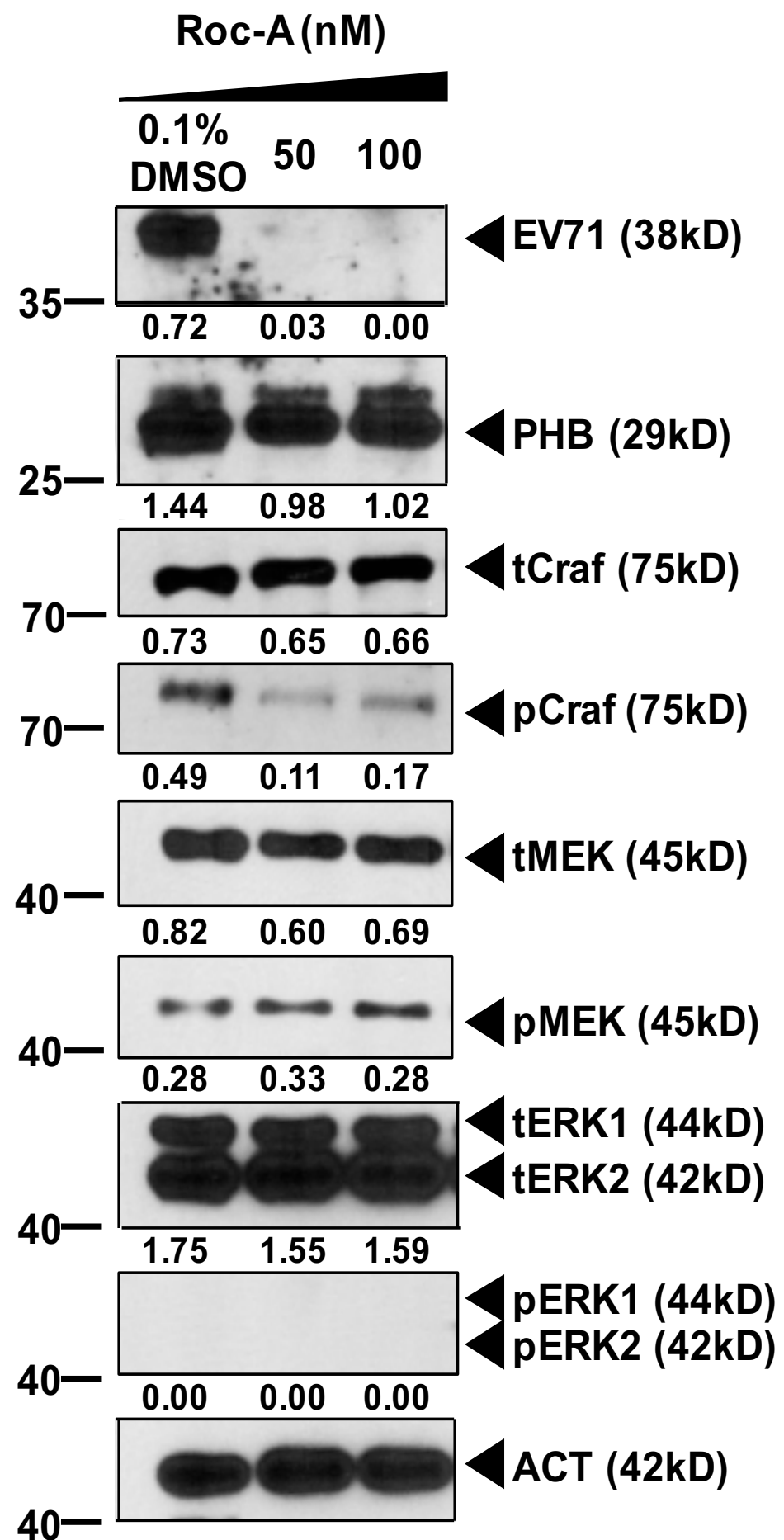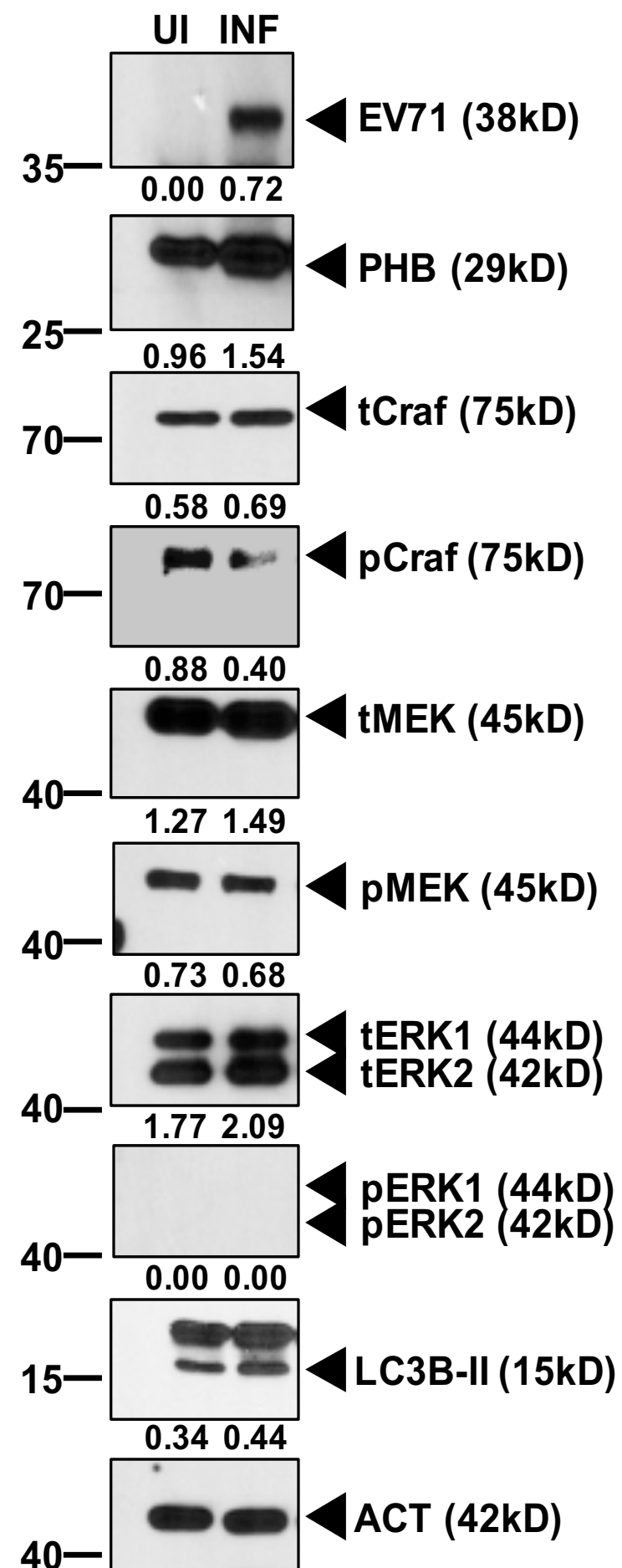

Supplement: S5 Fig — EV71-infected or uninfected NSC-34 cells were treated with Roc-A at concentrations of 50 and 100nM. At 48 hours post-incubation, the cell lysates were harvested and subjected to Western blot analysis. 0.1% DMSO-treated, uninfected and infected only cells served as control. UI, uninfected; INF, infected. One representative from two independent experiments is shown. (PDF) [file ppat.1006778.s012.pdf]

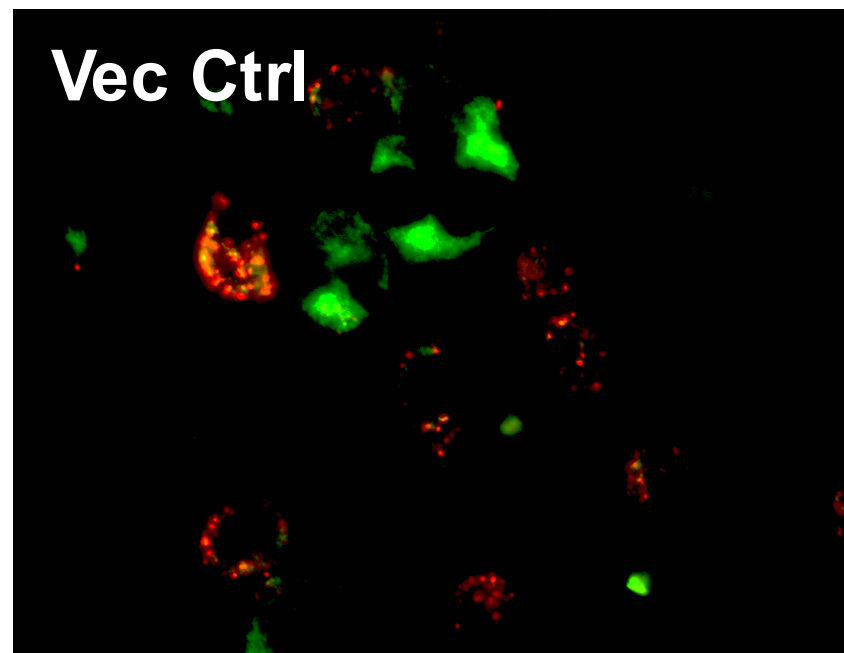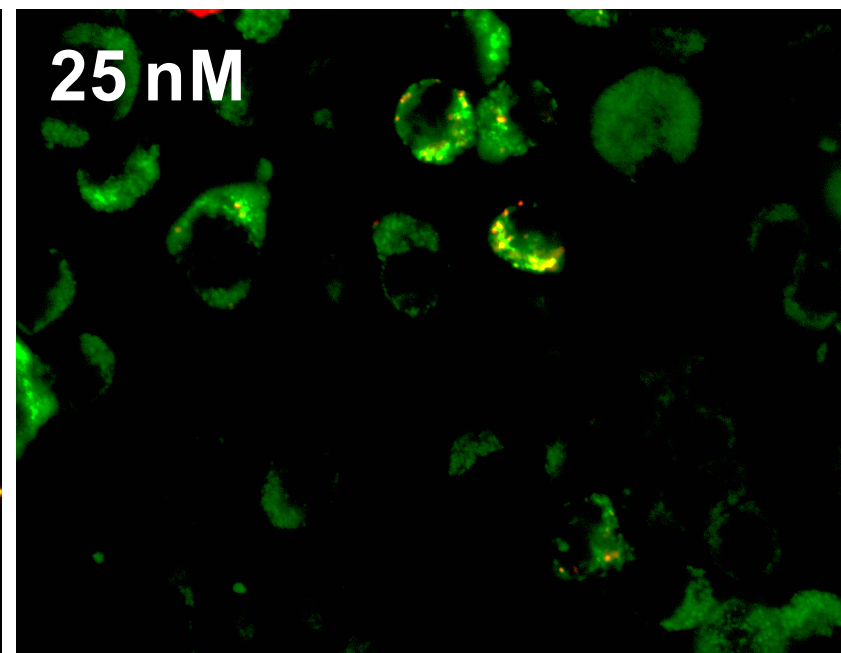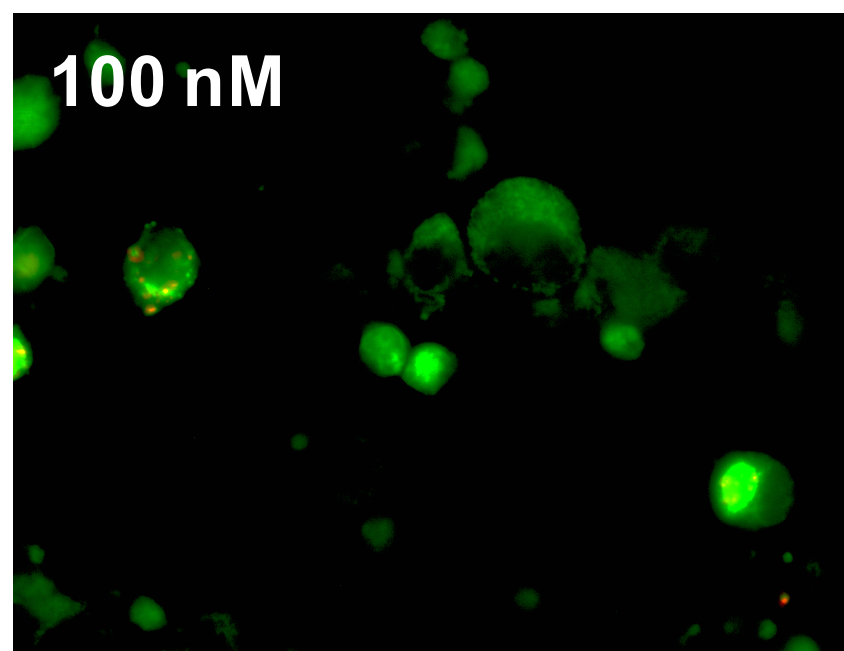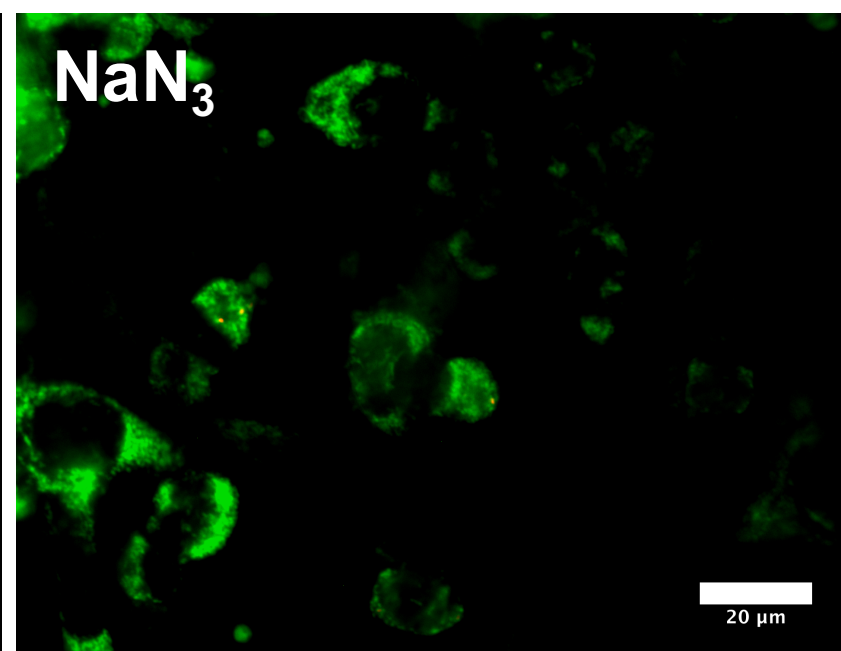

Supplement: S6 Fig — NSC-34 cells were incubated with various concentrations of Roc-A for 48 hours before assessment of mitochondrial membrane potential using JC-1 dye, with green-fluorescent monomer at depolarized membrane potentials or red-fluorescent J-aggregate at hyperpolarized membrane potentials (healthy mitochondria). Representative images were shown. Scale bar denotes 20 μm. (PDF) [file ppat.1006778.s013.pdf]

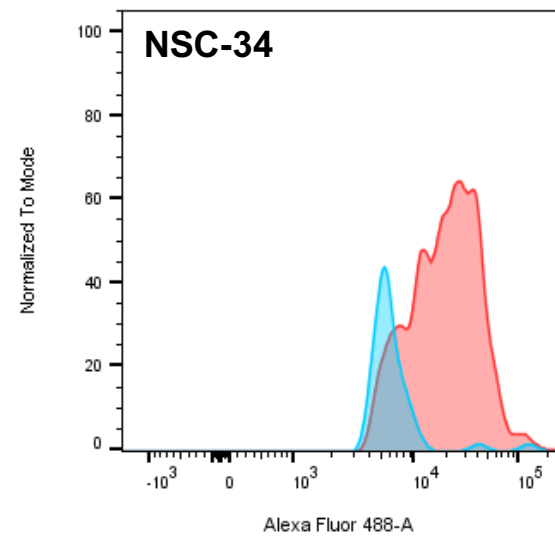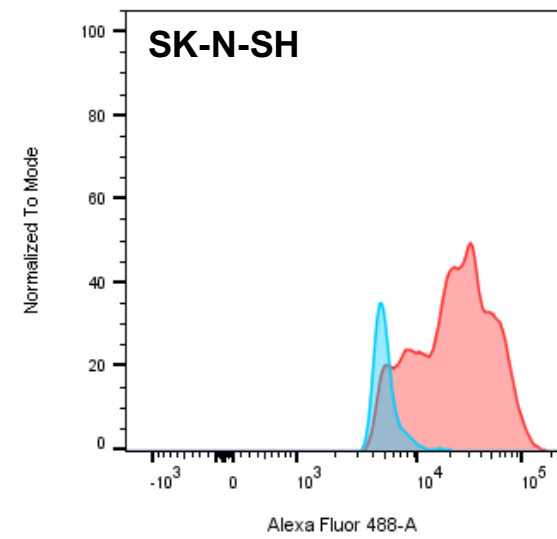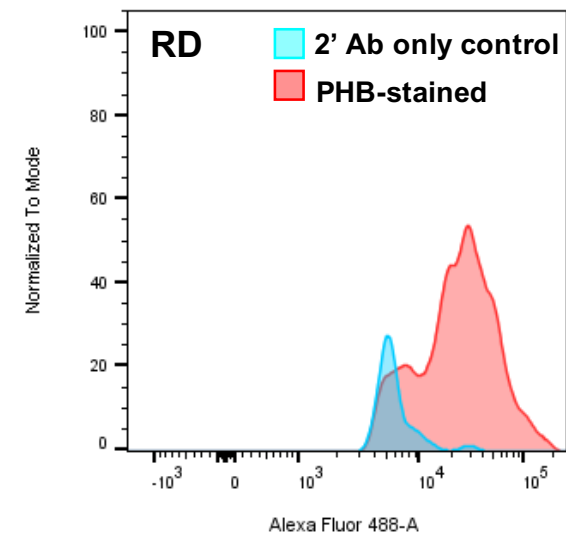

| Cell Lines | MFI   |
|------------|-------|
| NSC-34     | 25094 |
| RD         | 30859 |
| SK-N-SH    | 29211 |

Supplement: S7 Fig — Overlay histogram showing NSC-34, SK-N-SH and RD cells stained with anti-PHB antibody (red line). The cells were blocked with respective Fc blocker and stained with anti-PHB antibody or secondary antibody, prior to fixing with 4% PFA. Secondary antibody—stained cells (blue line) served as control. One representative of two biological repeats is shown. Median Fluorescent Intensity (MFI) values are shown for each cell line. (PDF) [file ppat.1006778.s014.pdf]

a

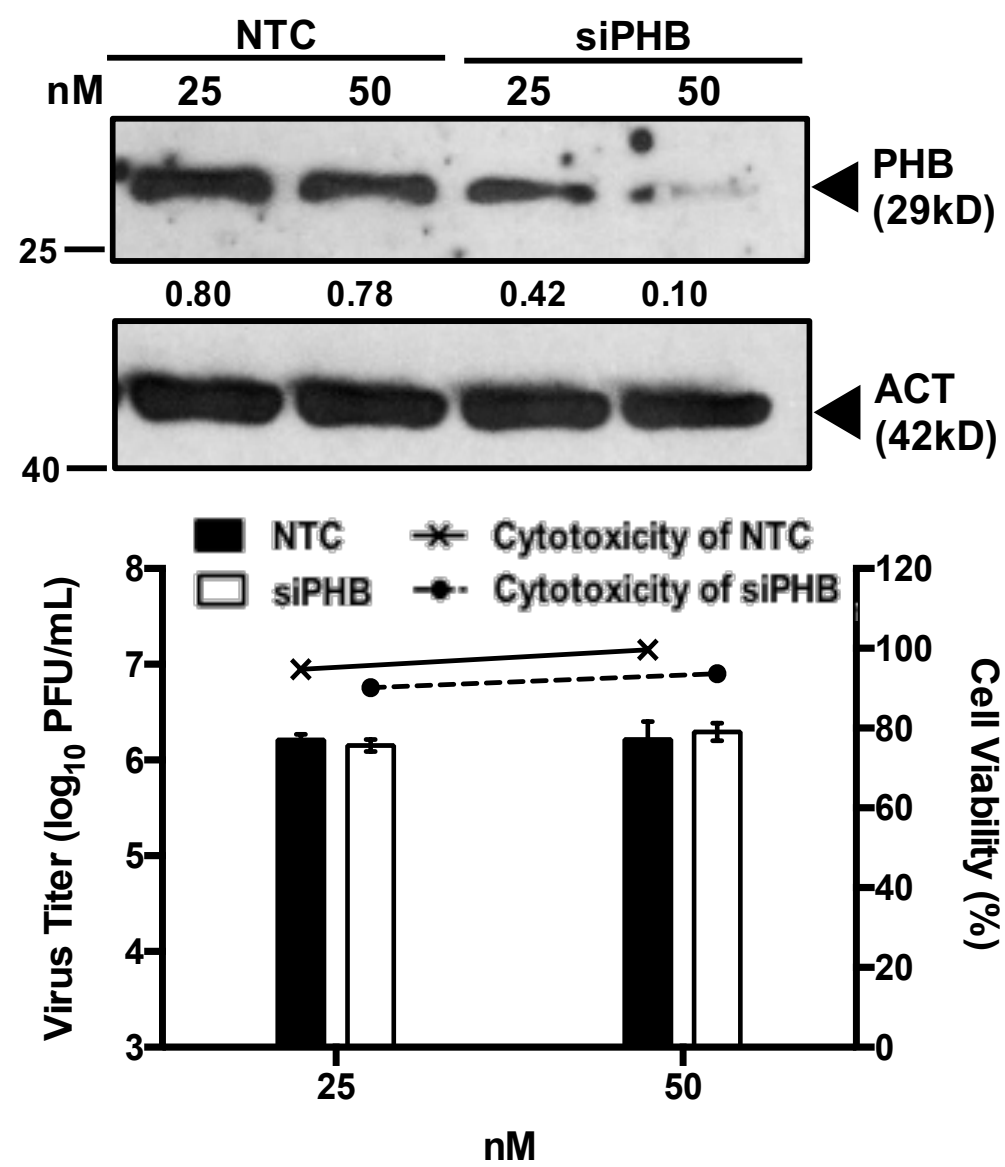

b

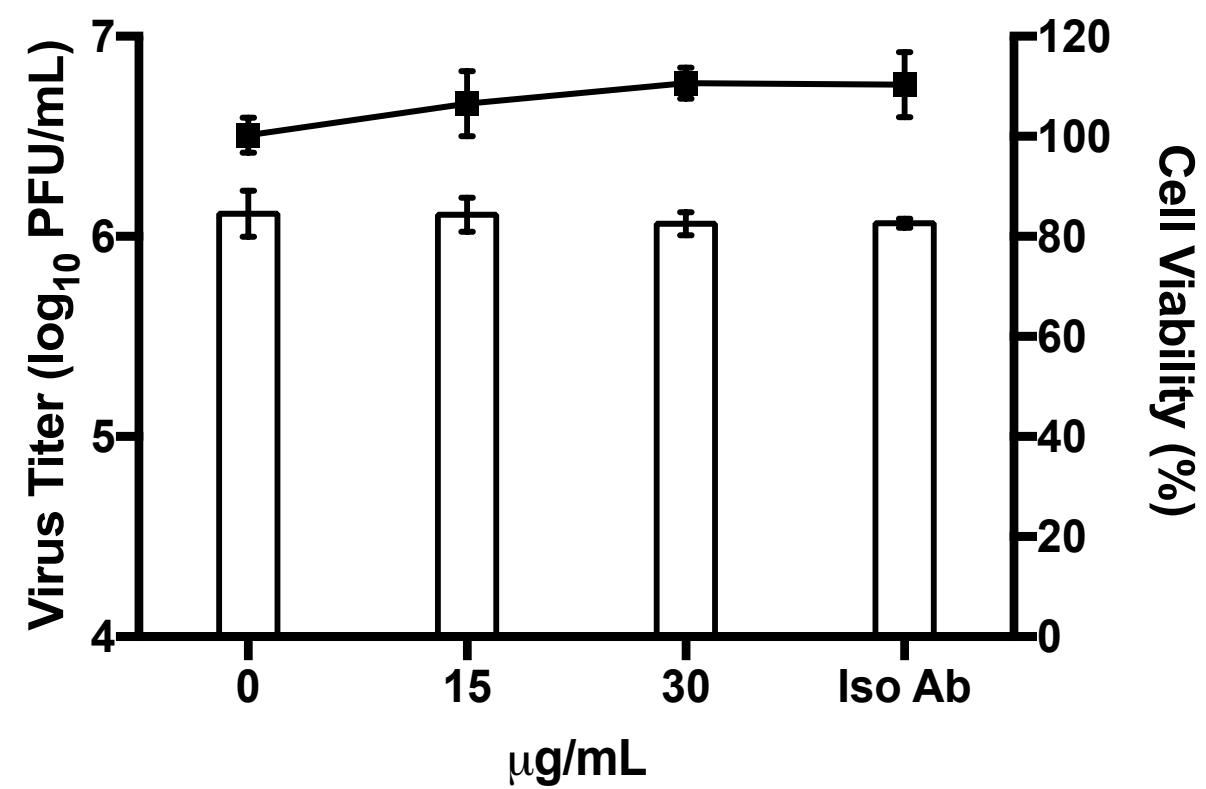

Supplement: S8 Fig — (a) RD cells were transfected with PHB siRNA at various concentrations. The efficiency of siRNA knockdown was confirmed by Western blot. PHB-knockdown cells were infected with EV71 S41 at M.O.I. 1. Viral titer in the culture supernatant was determined by plaque assay at 12 h.p.i. Statistical analysis was performed using two-tailed student’s t-test (* p<0.05, ** p<0.005, *** p<0.005, **** p<0.0001). Error bars represent mean ± standard deviation. Relative band quantification (below Western blot) was determined by ImageJ, by normalizing to loading control, β-actin. Non-targeting siRNA (NTC) served as control. (b) RD cells were pre-incubated with anti-PHB antibody or isotype control antibody for 1 hour before infection. Culture supernatant was harvested at 12 h.p.i. for viral titer determination. Cell viability was determined using alamarBlue cytotoxicity assay. One representative of two biological repeats is shown. (PDF) [file ppat.1006778.s015.pdf]
